# Modulation of the helical properties of DNA: next-to-nearest neighbour effects and beyond

**DOI:** 10.1093/nar/gkz255

**Published:** 2019-04-08

**Authors:** Alexandra Balaceanu, Diana Buitrago, Jürgen Walther, Adam Hospital, Pablo D Dans, Modesto Orozco

**Affiliations:** 1 Institute for Research in Biomedicine (IRB Barcelona), The Barcelona Institute of Science and Technology (BIST), 08028 Barcelona, Spain; 2 Department of Biochemistry and Biomedicine, University of Barcelona, 08028 Barcelona, Spain

## Abstract

We used extensive molecular dynamics simulations to study the structural and dynamic properties of the central d(TpA) step in the highly polymorphic d(CpTpApG) tetranucleotide. Contrary to the assumption of the dinucleotide-model and its nearest neighbours (tetranucleotide-model), the properties of the central d(TpA) step change quite significantly dependent on the next-to-nearest (hexanucleotide) sequence context and in a few cases are modulated by even remote neighbours (beyond next-to-nearest from the central TpA). Our results highlight the existence of previously undescribed dynamical mechanisms for the transmission of structural information into the DNA and demonstrate the existence of certain sequences with special physical properties that can impact on the global DNA structure and dynamics.

## INTRODUCTION

Early structural models of DNA derived from fibre diffraction data provide a static and averaged picture of the double helix (1–3), which despite its simplicity was sufficient to represent the general shape of DNA in physiological conditions. However, as more accurate structural techniques appeared, the intrinsic polymorphism of double-stranded DNA become evident (4–7) as significantly different conformations were described depending on the sequence, the environment or the presence of ligands (8–11). Six decades after the development of the first duplex models, we understand that DNA as a flexible and polymorphic molecule is able to sample a wide range of helical geometries, thanks to a complex choreography of backbone rearrangements, which allows the conformational changes required for DNA functionality (11–19).

Attempts to determine the principles relating sequence and structure originated in the eighties when by processing the scarce experimental data available, Calladine *et al.* (20), developed a series of heuristic rules relating sequence with some structural characteristics of DNA (21,22). In the late nineties (23), Olson *et al.* developed a complete set of parameters defining the expected distribution of helical parameters of the 10 unique base pair steps (bps). Parameters were derived from the analysis of the available crystal data on DNA–protein complexes and provided information not only on the equilibrium geometry but also on the expected flexibility of the bps (extracted from the variability of the same bps in different crystals). Twenty years after their generation, Olson-Zhurkin parameters are still used to represent DNA by means of helical mesoscopic descriptors. However, we cannot ignore the strong assumptions involved in their derivation: (i) the ensemble of configurations obtained from the analysis of crystal structures should define a densely populated Gaussian distribution; (ii) a dinucleotide (step) model is enough to represent DNA sequence variability, i.e. the helical geometry can be decomposed at the bps level; (iii) conformational variability found in structures in PDB should exclusively depend on the flexibility of the step and finally (iv) binding of a protein should not introduce anharmonic distortions in the duplex geometry.

The eruption of atomistic molecular dynamics (MD) simulations gave the community an alternative source of parameters to describe DNA structure and flexibility. Compared with results derived from the analysis of experimental structures, the MD-based ones are more robust as they are obtained from processing an extremely large number of snapshots, and provide information on flexibility that is not contaminated by the presence of ligands, crystal lattice or any other environmental artifacts. As a major caveat, MD-derived descriptions of DNA properties are dependent on the length of trajectories as well as on the quality of the force field parameters used to describe DNA interactions. Thus, early attempts to describe DNA from multi-nanosecond trajectories led to artefactual results due to a previously unknown error of the most used force field at that time (24). A newer force field (25) and higher computational capabilities provided descriptions of DNA properties that were more reasonable, but still far from the required accuracy (12,26,27). The availability of the highly accurate PARMBSC1 force field (28,29) and the development of new MD codes taking advantage of a new generation of computers (30–33) provide the community with the possibility to derive reliable representation of the sequence-dependent physical properties of DNA from the analysis of microsecond long trajectories collected under highly controlled simulation conditions.

Results collected by the Ascona B-DNA Consortium (34–37) revealed two major findings that challenged current models of DNA flexibility. First, the dinucleotide-model is insufficient to describe DNA flexibility, as the variability in bps parameters depending on tetranucleotide environment can be more pronounced than the variability found when comparing different bps for a given tetranucleotide context. Second, several distributions of helical parameters considering the nearest neighbours deviate from normality and a part of them are in fact multi-modal, which means that the physical properties of such tetranucleotides cannot be represented by a single set of elastic parameters (equilibrium values and associated stiffness). Analysis of MD data revealed that the changes between substates happen towards a series of coordinated changes along the backbone (17,37,38), where unusual H-bond interactions and subtle changes in the solvent environment play a key role (18,39). The analysis of ABC data and of additional trajectories stored in our BigNASim database (40) suggested that a nearest neighbour-based model was in general sufficient to derive transferable descriptors of DNA structure and flexibility, but a few exceptions to this general rule emerged; the clearest one is the d(CpTpApG) tetranucleotide (in the following CTAG): a very polymorphic stretch of DNA, with 50% G-C content, for which results were significantly different depending on the simulation. The structural peculiarities of TpA steps have been qualitatively pointed out in the past by analysing a small number of experimental structures, especially when immersed in short A-tracks (41,42).

We present here a detailed analysis of CTAG in different sequence contexts. Results demonstrate that next-to-nearest effects modulate the geometrical properties of the central d(TpA) step. Such structural effects are very visible when hexanucleotides are considered, but quite surprisingly extend beyond the next-to-nearest level, indicating the existence of a complex mechanism of information transfer across DNA through the coordinated backbone and base movements.

## MATERIALS AND METHODS

### The choice of sequences and the simulation details

The systematic study of sequence-dependent effects beyond the tetranucleotide level has been to date impossible, due to the huge number of sequences that need to be considered. For example, the study of all hexanucleotides would require the simulation of 2,080 sequences, while to consider all octanucleotides 32,826 sequence combinations are needed. Fortunately, the analysis of ABC simulations where tetranucleotides appear in different molecular environments suggests that sequences effects beyond the tetranucleotide are rare, and if they exist, are localized in certain ultra-flexible sequences. We focused our interest here in one of the most flexible tetranucleotide: CTAG. Thus, we built a library of 40 different sequences covering the entire hexanucleotide space (XpCpTpApGpX) as well as all possible pyrimidine(Y)/purine(R) combinations at the octanucleotide level in several repeats (see Supplementary Methods). All the sequences were prepared using the leap module of AMBERTOOLS 16 (43) and standard ABC protocol (37). Accordingly, systems were built from Arnott’s B-DNA average parameters, neutralizing the DNA with K^+^ ions, adding water (at least 10 Å of water separate DNA from the faces of the box) and extra 150 mM KCl. Systems were then optimized, thermalized and equilibrated before production (34,35). Water was represented with the SCP/E model (44), Smith-Dang parameters were used for ions (45–47) and the recent PARMBSC1 force field was considered to represent nucleic acids interactions (28). Trajectories (collected in the NPT ensemble *T* = 298 K, *P* = 1 atm) were extended from 0.5 μs to up to 9 μs. All simulations were performed with the pmemd.cuda code using periodic boundary conditions and Particle Mesh Ewald (31,48). Movements of hydrogen atoms were annihilated using SHAKE (49), which allowed us the use of a 2 fs integration step. All trajectories collected here are accessible through the MuG BigNASim portal (40): https://mmb.irbbarcelona.org/BIGNASim/

### Analysis

Standard analysis was done using *cpptraj* module of the AMBERTOOLS 16 package (43), the NAFlex server (50) CURVES+ and CANAL programs (51), following the standard ABC-conventions (37). The CANION module from Curves+ (38) was used to determine distributions of ion populations in curvilinear cylindrical coordinates for each snapshot of the simulations with respect to the instantaneous helical axis. Duplexes were named following the Watson strand (e.g. ATGG stands for (ATGG)·(CCAT)). The letters R, Y and X stand for a purine, a pyrimidine or any base respectively, while X:X and XX represent a base pair and base pair step, respectively. Base pairs flanking the CTAG were denoted using two dots to represent the central tetrad (e.g. R··Y). The normality and modality of the helical distributions were evaluated using Bayesian Information Criteria (52,53) and Helguerro’s theorem (54) as described elsewhere (12). Classification of the torsional states of the different rotatable bonds in the DNA backbone was done using standard criteria (55). Correlations between different torsions were determined by circular correlation analysis (see Supplementary Methods for additional details). The meta-trajectory analysis was used to define the global characteristic of the d(TpA) essential deformation space. With this purpose, the 40 individual trajectories were grouped and subjected to principal component analysis (56,57) in the helical space of the central d(TpA) step after Lankaš’ normalization of the different rotational and translational degrees of freedom (58). The essential dynamics of the central d(TpA) step is then used to define the set of key movements explaining the global deformation at the d(TpA) step. The distributions of the four informative bps deformations were subjected to detailed analysis (see Supplementary Method for additional details). Comparison and clustering of the individual trajectories of the central d(TpA) for the 40 sequences studied (all with a common CTAG central tetranucleotide) were done using symmetrized Kullback-Leibler (KL) divergences (58) followed by hierarchical cluster analysis using Ward’s clustering criterion (59), where the dissimilarities are squared before cluster updating (60), using as descriptive variable the six distinguished helical variables detected by the PCA of the meta-trajectory (see Supplementary Methods for additional details). The clusters obtained in this manner were subsequently analysed in detail, further highlighting the differences between their individual accessible helical spaces. Ion analysis was performed as described elsewhere (18,38) to unravel the connections between the binding of cations on the DNA and its mechanistic properties. Stacking strengths were followed by geometrical criteria for the central dinucleotide in the meta-trajectory filtered by the three main states in helical space, as described in detail in Supplementary Methods. Structural database analysis was done using all DNA structures containing the CTAG tetranucleotide. Genomic analysis was done to determine the prevalence of the CTAG tetranucleotide in different wild-type genomes and its resilience to mutation. Genomes of *Homo sapiens*(hg19), *Escherichia coli*(NC_000913.3) and *Saccharomyces cerevisiae*(sacCer3) were analysed. Occurrences of this tetranucleotide were then mapped, using Homer software (61), to the annotated regions of each organism obtained from UCSC and compared to the overall frequency of each annotation type. To compute the resilience to mutation, the frequency of mutations for each tetranucleotide along the genome in 30 different cancer types (data from (62)) was determined normalizing by tetranucleotide occurrence along the genome. Single-nucleotide polymorphisms (SNPs) in the human genome were retrieved from Ensembl Variation database (63), and the number of SNPs per tetranucleotide was computed, normalizing by genome-wide tetranucleotide frequency.

## RESULTS AND DISCUSSION

### The CTAG shows dramatic and complex structural polymorphism

We collected trajectories for 40 oligonucleotides containing the CTAG tetranucleotide in a central position (see ‘Materials and Methods’ and Supplementary Table S1). All the trajectories were stable along time in the sub-microsecond timescale, sampling structures that fit well in the B-like double helical conformation. As suggested by the analysis of ABC-simulations (37), and of trajectories deposited in BigNASim, (40) CTAG is highly polymorphic as seen from clear bimodal distributions of some helical parameters. To check that the multi-peaked distributions were not artefacts due to limited sampling, we extended trajectories for selected tetranucleotides up to 9 μs (Supplementary Table S1), tracing the changes in the distribution of helical parameters. The good convergence shown in Supplementary Figure S1 supports the robustness of our results and suggests a fast dynamic of interchange of the different states (see ‘Discussion’ section below).

**Figure 1.**
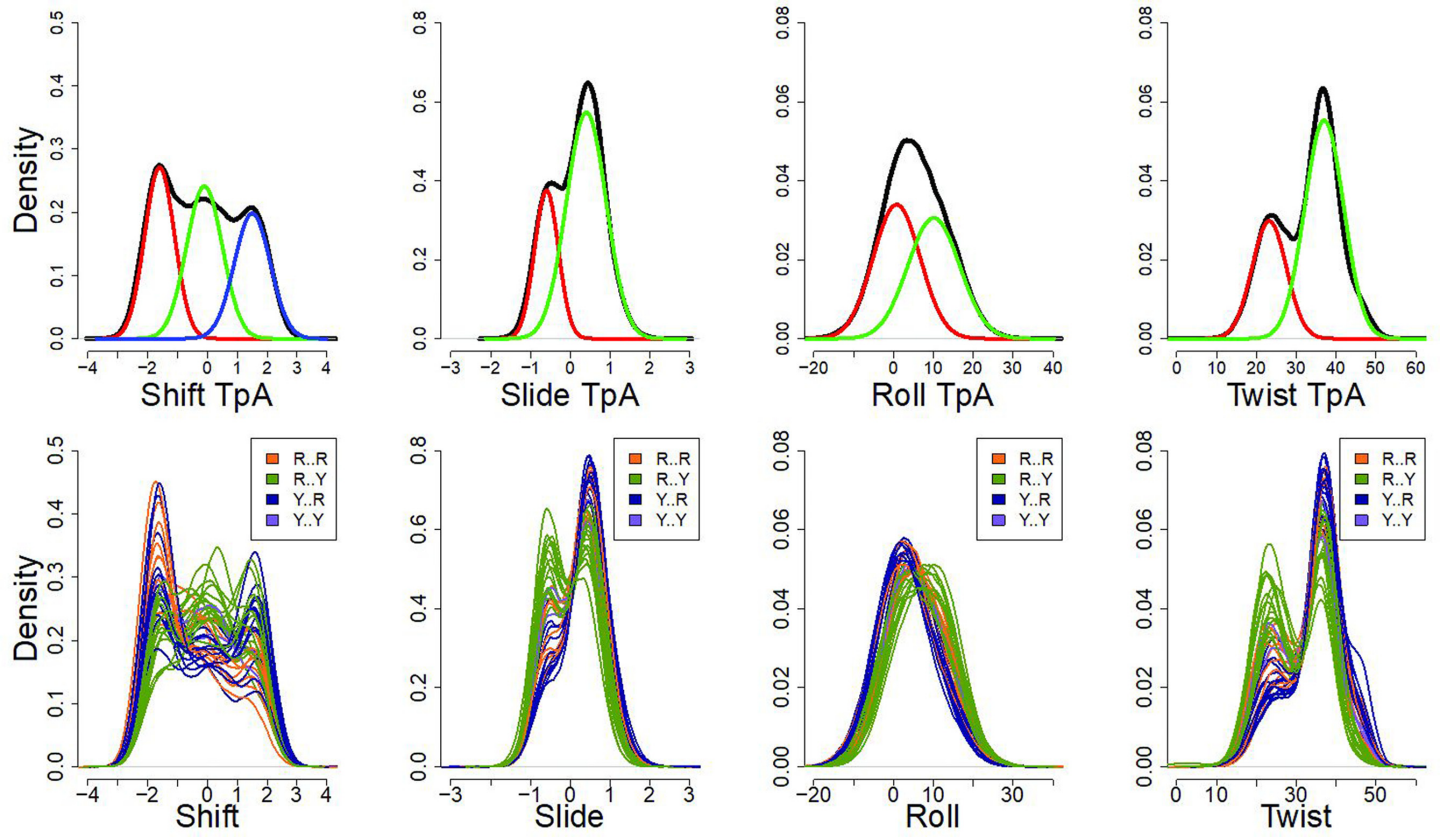
Normalized frequencies of those bps helical parameters found to be bi-normal and tri-normal according to the BIC analysis. First row: Density obtained from the meta-trajectory (black line), and the BIC decomposition in two Gaussians (slide, roll and twist: red and green lines) or in three Gaussians (shift: red, green and blue lines). Second row: Overlapped density of the shift, slide, roll and twist parameters at the central TpA step of the 40 sequences studied (see Supplementary Table S1).

**Figure 2.**
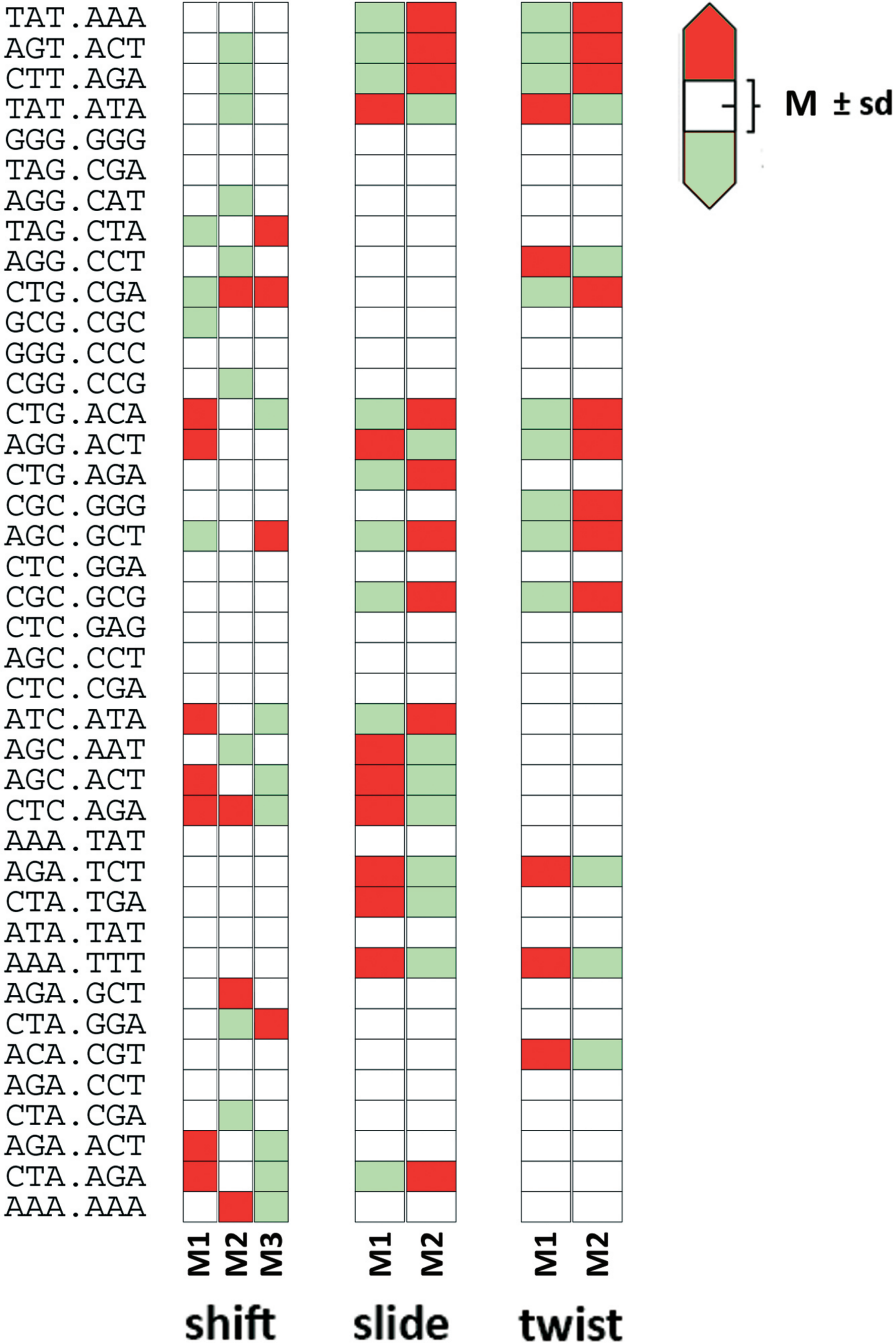
Relative propensities of the multi-modal bps helical coordinates of the central TpA in all 40 sequence contexts. Comparison to the global average propensities over all sequence contexts per component of the multi-modal distributions with standard deviations that reflect the variation of the propensity of each component amongst sequences. The propensity values were computed BIC analysis (see ‘Materials and Methods’ section and Supplementary Methods).

In order to obtain a global average picture of the conformational space accessible to the CTAG tetranucleotide, we joined the 40 individual trajectories (equal number of snapshots in all cases) to generate a meta-trajectory, which was then subjected to PCA and BIC analysis. Four base-parameters (the symmetric buckle and propeller twist of d(T·A) and d(A·T)) and four bps parameters at the central d(TpA) step (roll, twist, shift and slide) emerged as determinant to explain 60% of the variance in the meta-trajectory; Six of which were used for further analysis. As seen in the BIC analysis summarized in Figure 1, deviations from Gaussianity in the form of multi-peaked distributions are the main responsible for the structural polymorphisms detected at the bps level. Such deviations could in principle emerge from two different sources: (i) intrinsic multi-modality in the individual trajectories and (ii) individual distributions (coming from the 40 sequences studied) are Gaussian, but they are centred at different average values. To analyse which is the real origin of the deviation from normality in meta-trajectories, we repeated the analysis for individual trajectories (Figure 1). Roll distributions were unimodal in all cases, but the position of the peak was displaced towards slightly higher values when the central tetranucleotide is surrounded by R at 5′ and Y at 3′ (i.e. RpCpTpApGpY hexanucleotides), leading to a bi-normal distribution of the meta-trajectory (see Figure 2). The situation is completely different for twist, slide and shift where bi- or even tri-modality (three peaks in the distribution) is clear for individual sequences (see Figure 2 and Supplementary Figure S2), with the different substates being sampled in a fast equilibrium along the time scale of the simulations (see examples in Supplementary Figure S3).

**Figure 3.**
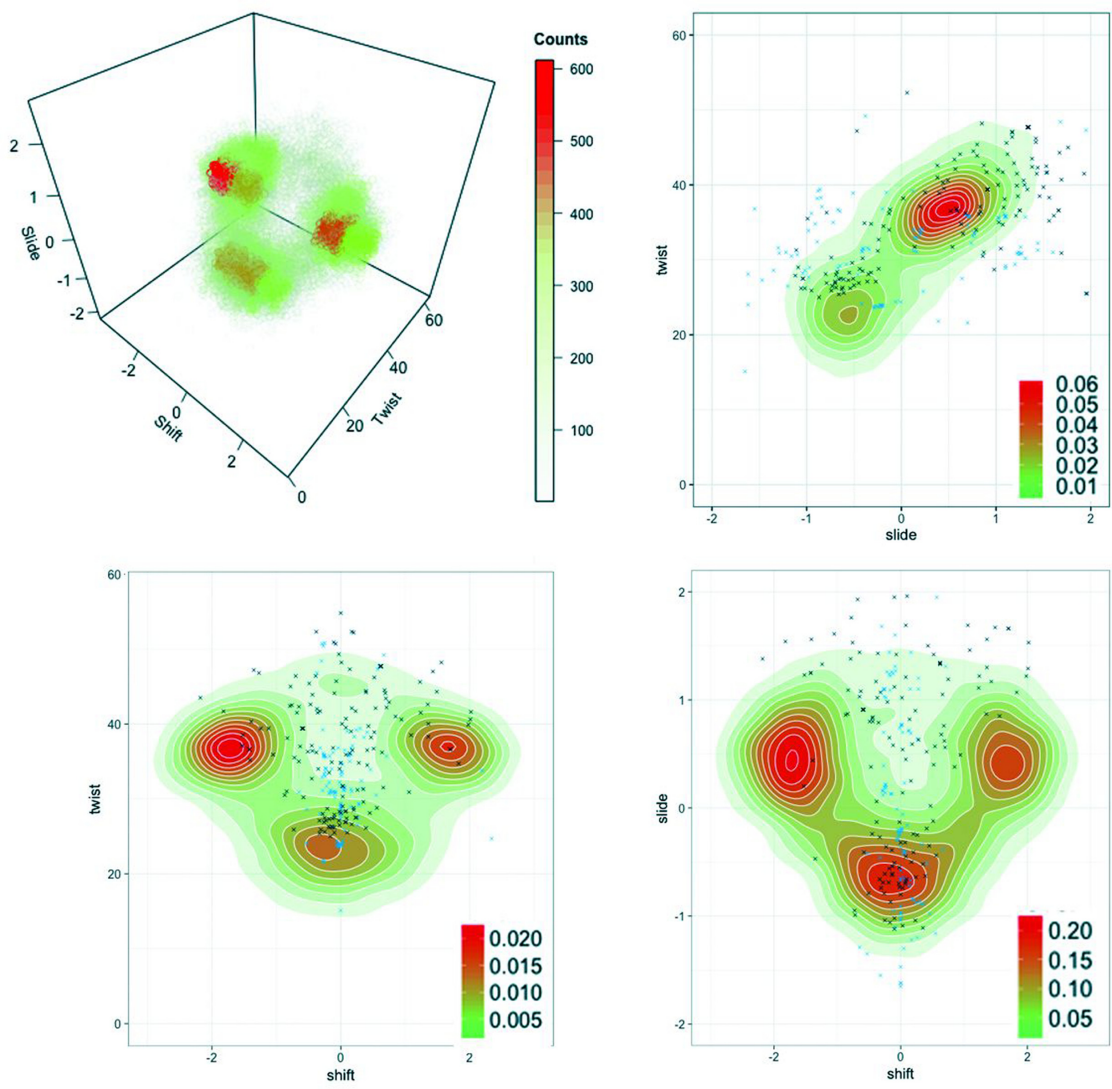
3D and 2D counts in the shift, slide and twist planes from MD simulations at the central TpA step. In the 2D density plots, experimental structures from the PDB (see Supplementary Methods) were added as black crosses (protein–DNA complexes) or blue crosses (isolated DNA).

**Figure 4.**
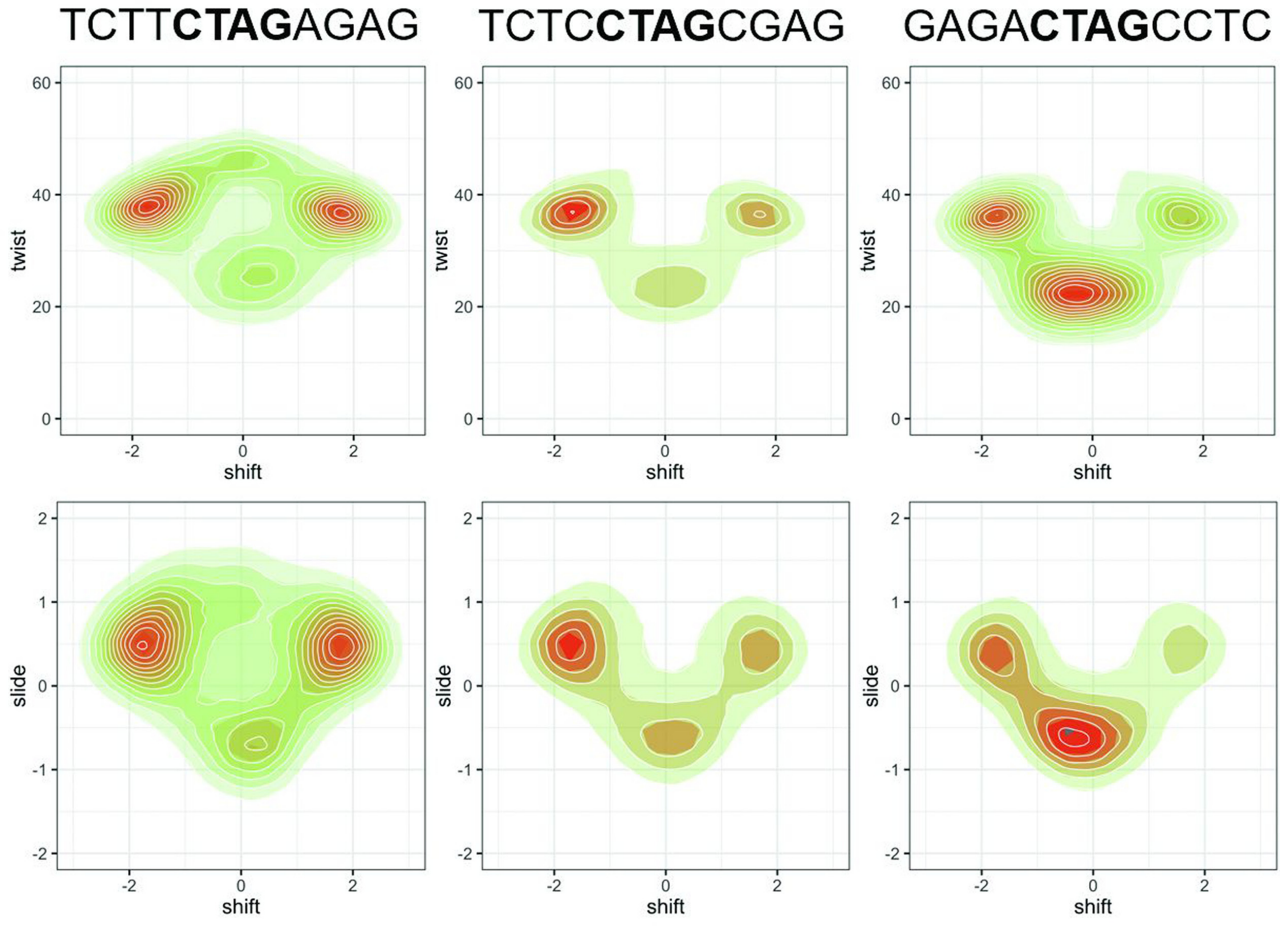
2D density plots in the shift/twist and shift/slide planes at the central TpA step for three selected sequences.

As shift distribution is tri-modal and twist and slide distributions are bi-modal, we could in principle expect 12 states. However, many of the combinations of twist, slide and shift substates are not possible, and in practice, only four states appear when meta-trajectory is projected in the twist-slide-shift 3D space (Figure 3). In fact, one of them (high twist/positive slide/zero shift; HPZ) is populated only in some of the simulations and has globally a reduced impact in the meta-trajectory ensemble, which is dominated by three main states (Figure 4): high twist/positive slide/negative shift (HPN); high twist/positive slide/positive shift (HPP) and low twist/negative slide/zero shift (LNZ). Experimental validation of the suggested polymorphisms is difficult as experimental structures are always averaged (i.e. assuming a normal unimodal distribution). However, plotting the scarce experimental data available for the CTAG tetranucleotide on the 2D population plots (shift-twist, shift-slide and twist-slide) derived from meta-trajectories provides an indirect, but strong support to our results. For example, the shift distribution is very narrow and centred around zero for low slide values, while when slide increases, larger values (either positive or negative) of shift are sampled, in perfect agreement with MD meta-trajectories. Similarly, low twist appears experimentally only in zero shift conformations, while high shift (either negative or positive) is found only in experimental structures with a high twist. Although the major discrepancies between MD and experiments seem to occur for the twist-shift plane, filtering the shift values according to low/high twist reconcile partially the matching between experiments and theory (Supplementary Figure S4). Finally, the twist-slide plot shows only two regions of high probability consistent with the same slide/twist correlation found experimentally (see Figure 3 and ‘Discussion’ section below).

### Next-to-nearest dependence in central d(TpA) conformation

**Figure 5.**
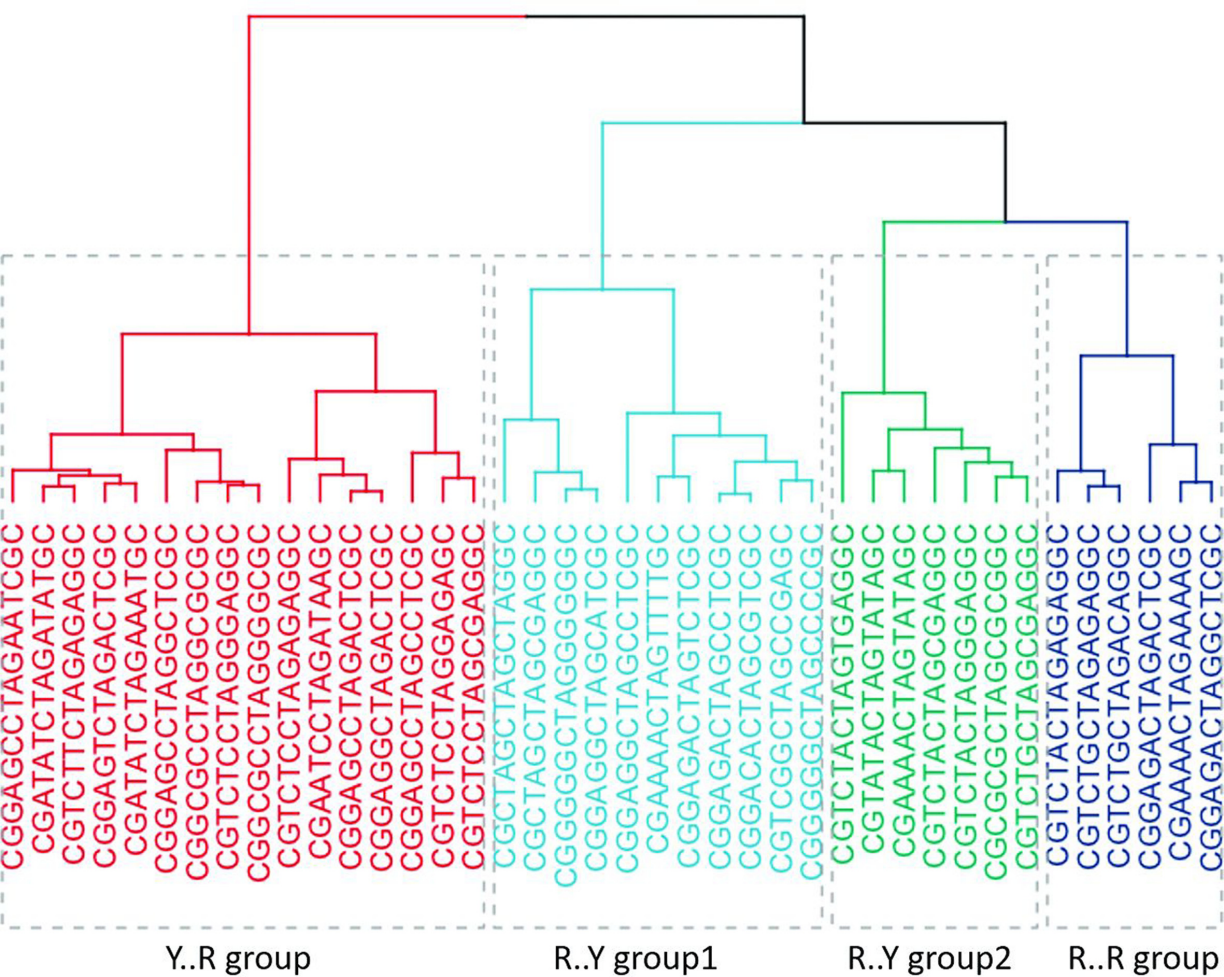
Dendrogram obtained from a hierarchical clustering method using Ward’s criterion to classify the sequences. The distances were obtained from the symmetric Kullback-Leibler (KL) divergence in the space of six helical parameters: shift, slide and twist of TpA step, buckle and propeller of dT, and the buckle of dA (see Supplementary Methods).

All the sequences studied here correspond to the same tetranucleotide, so a similar distribution of helical parameters at the central d(TpA) step could be expected. However, this is not the case as shown in selected examples in Supplementary Figure S2, where large differences in the distributions of helical coordinates for the d(TpA) step appear. Analysis of the trajectories (Figure 1) reveals that the origin of the difference emerges from the different weights of the individual substates defining the global distributions (see a global summary in Figure 2). Moreover, we observe that the varying populations of these substates are a direct consequence of sequence context. To go deeper in the analysis of this hexanucleotide variability, we perform Kullback-Leibler (KL) analysis of the 40 trajectories in the 6D space defined from the PCA analysis as informative of the entire flexibility space of the helix (see above). Clustering analysis can be performed from the KL results to determine the similarity between sequences based on the dynamics of the central d(TpA) step and organized in the relational dendrogram (Figure 5), which clearly shows the presence of at least two major clusters. The first one is populated mainly by sequences where the central tetranucleotide is flanked by Y at 5′ and R at 3′, but also contains two 5′Y·3′Y sequences. The other cluster, the largest one, is subdivided into three different subclusters, two of which are formed almost exclusively of sequences where the central tetranucleotide is surrounded by R at 5′ and Y at 3′; finally, the last cluster corresponds to situations where the CTAG tetrad is surrounded by 5′R··3′R. Examples of prototypical distributions obtained for representative sequences in each cluster are shown in Supplementary Figure S5, which demonstrate that the hexanucleotide content has a non-negligible role in defining the properties of the central d(TpA) step in the CTAG tetranucleotide, a clear exception of the nearest neighbour model. Furthermore, the presence of some hexanucleotides in different clusters suggests that some couplings might be possible even beyond the next-to-nearest neighbour level (see below). The rules that govern the sampling of a given substate of the sequences in each cluster can be understood by analysing sequence-dependent stabilizing factors that give rise to the characteristic distributions of helical parameters depicted in Supplementary Figure S5.

**Figure 6.**
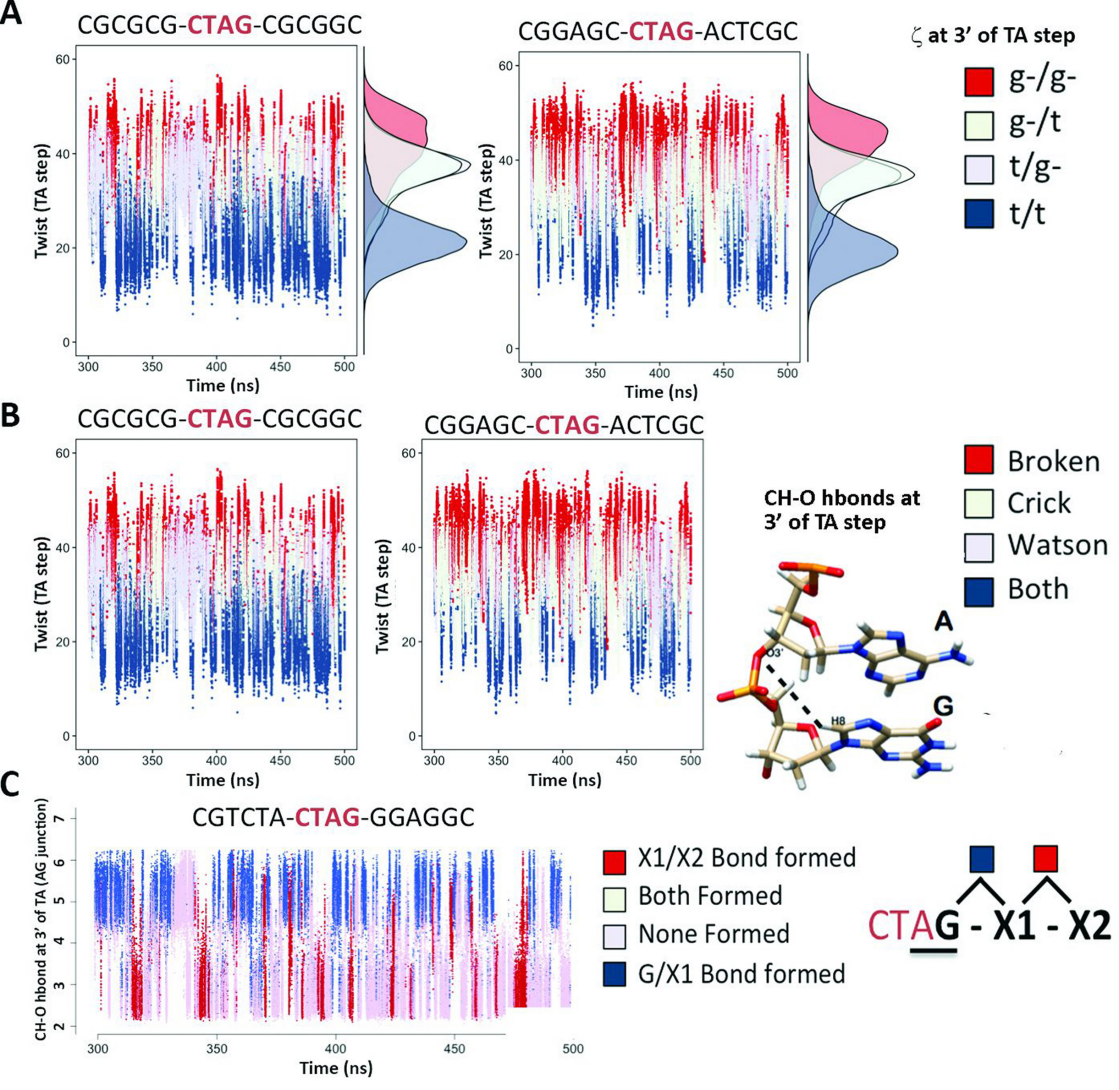
Concerted movements along the backbone and the bases explain the flow of structural information from the central TpA step and beyond next-to-nearest neighbours. (**A**) Correlation between twist and the BI/BII population (reduced to the ζ torsion at the 3′-side of TpA) at the TpA junction. (**B**) Correlation between twist at TpA and the CH-O h-bond formed at the ApG junction (bps +1). (**C**) Correlation between the CH-O h-bond at the ApG junction with the CH-O h-bond at bps+1 (hexanucleotide context) and bps+2 (octanucleotide context). Note that the CH-O h-bonds are always coupled to BII propensities, stabilizing the BII substate.

The existence of such effects implies that the motion of the central TpA step must be somehow connected to the distant base pairs. Mechanical information should travel from one site to the other to allow the TpA step to ‘feel’ its environment and respond in a different way according to the nature of the base pairs located almost half helical turn away. We were able to find a possible explanation based on the concerted and correlated movements of the backbone and bases, by first noting that the twist polymorphism at TpA was behaving as the better well-known YpR step: d(CpG) (18,34,37,39). The two possible twist substates (HT/LT) at the TpA step were connected to the backbone BI/BII polymorphism at the next GA junction (note that BI/BII interconversion is mainly governed by the ζ torsion). Furthermore, the BI/BII polymorphism at GpA is possible due to the formation of the intra C8H8-O3′ h-bond and the shift polymorphism in the same junction (Figure 6A and B) (39). Similar results were found if looking to the correlation of twist at the central TpA step with the bps at the 5′-side (CpT). It is then clear that the main backbone polymorphism (BI/BII) is linked to the base polymorphisms, mainly to shift and twist (Supplementary Table S2) up to the next-to-nearest neighbours. The information travels through successive backbone and base polymorphisms, which are limited to some specific substates due to DNA’s crankshaft motion (Supplementary Table S2). This dynamically concerted movement of either (alone or in combination) shift/slide/twist step parameters and the ζ torsion could be appreciated from the Pearson correlation coefficients that clearly show a correlation/anti-correlation pattern in successive bps. Since intra-molecular CH-O h-bonds are mainly responsible for the information transfer between the backbone and the base (39) (with perhaps a small contribution from the known sugar puckering flexibility, see Supplementary Table S2), both backbone and base polymorphisms can be followed by looking only to the formation of those C8H8-O3′ hbonds in RpR and YpR steps, or C6H6-O3′ hbonds in RpY and YpY steps. The correlated/anti-correlated formation of these h-bonds away from the central TpA step clearly explains the transfer of mechanical information up to the next-to-nearest neighbours, and also beyond depending on the sequence (see ‘Discussion’ section below and Figure 6C). As a general rule, at the tetranucleotide level, the BII backbone state is significantly favoured at the 3′ side on either strand (i.e. at GpA step). The correlations of backbone substates with the helical parameters at TpA paint a picture where negative shift is related to having more BI at the GpA of the Watson strand and more BII at GpA on the Crick strand, with positive shift being favoured in the exactly opposite situation. Additionally, the TpA can be found in a low twist state only when both 3′ GpA junctions are in BII, while the simultaneous BI state on both strands at GpA will promote high twist at TpA. The next-to-nearest context and sometimes more remote sequence effects can modulate the relative populations of BI/BII on the two strands, which in turn will affect the helical parameters at the central TpA. It’s worth noting that the correlations between helical parameters in consecutive steps are mostly anti-correlations, and in general the global twist distribution of a tetra- or hexanucleotide segment can be perfectly described by a single Gaussian function. This means that, from a static and averaged view, the correlations/anti-correlations between substates in consecutive steps are leading to compensatory effects.

In addition to the backbone movements and h-bonds, each substate at the TpA step is modulated and stabilized by other factors, such as interactions with ions and stacking between consecutive bases. For CpG, a relatively simple mechanism was found where the entrance of Na+/K+ inside the minor groove triggered and stabilized the low twist state and hence BII (18). For TpA, the mechanism is much more complex, since it involves a combination of shift/slide/twist substates and the movements of K+ from the major groove of CpT to the major groove of ApG, when going from HPN (high twist/positive slide/negative shift) to HPP (high twist/positive slide/positive shift) (Supplementary Figure S6). A depletion of cations inside both grooves for the whole tetranucleotide was observed when moving to the LNZ substate (low twist/negative slide/zero shift). All the sequences studied share the same redistribution of K+ when moving between the substates, but the sequence-specific populations of each substate lead to different overall ion distributions when changing the next-to-nearest neighbour’s context (Supplementary Figure S7). Finally, we found that at the TpA step, the stacking strength on either strand increased significantly when shift moves toward the minor groove at high twist and positive slide, an interaction that further stabilizes the BII state at the 3′ junction (Supplementary Figure S8).

### Structural information travels beyond next-to-nearest neighbours

**Figure 7.**
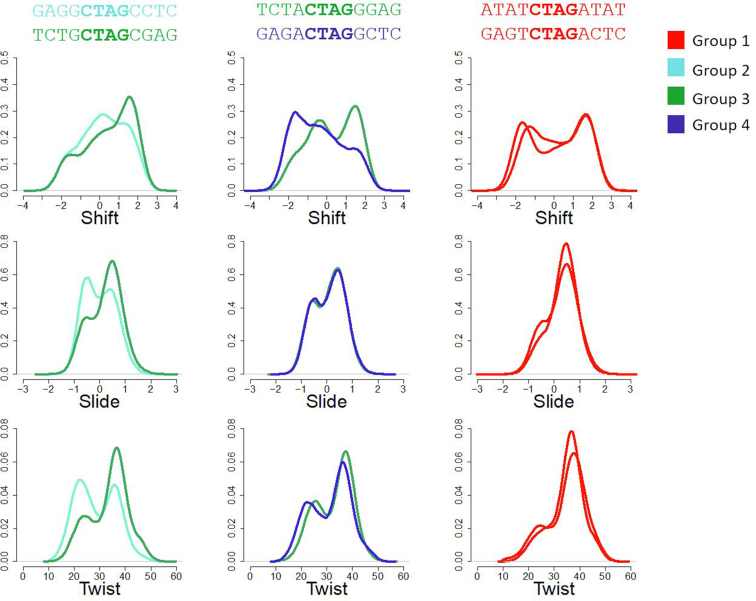
Normalized frequencies of shift, slide and twist at the central TpA step for three pairs of selected sequences showing non-negligible effects beyond next-to-nearest neighbours. The colours used are related to the groups found in the clustering analysis.

Sequences studied here cover all the next-to-nearest neighbours’ space with some redundancy that allowed us to check for some remote effects beyond this level. As noted above, such effects are clearly visible already in Figure 5, where sequences containing the same hexanucleotide sequence appear in two very different branches of the dendrogram, indicating the tuning of hexanucleotide preferences by more remote effects. Analysis of the different octanucleotidic environments (R··R/Y··Y), (Y··R) and (R··Y) reveals the existence of a quite differential behaviour (see Figure 7). For example, the conformational substates of the central TpA step in YpCpTpApGpR sequences (Y··R) are fully defined at the next-to-nearest neighbours level, with remote effects being negligible: all (Y··R) hexanucleotides appear in the same cluster in the dendrogram of Figure 5, and they display consistent distributions in all multi-modal helical parameters (shift has two main populations at ±2 Å, with the zero shift state being less favoured). Slide and Twist are, as a consequence, pushed towards higher values. This makes sense, considering that, irrespective of the octanucleotide level base, when ApG is followed by an R base on both strands, the junction at ApG will be pushed out of the BII state. This frustration of high BII propensity of two adjacent bps (a direct consequence of the crankshaft effect) will result in an overall higher BI population at ApG, which corresponds to the high twist, positive slide and negative/positive shift equilibrium at TpA. On the contrary, R··Y hexanucleotides (RpCpTpApGpY sequences) have two very distinct behaviours depending on the next flanking base: Central TpA steps in RpRpCpTpApGpYpY (RR··YY) octanucleotides tend to populate zero shift states and have equal populations of high/low twist as well as of negative/positive slide. On the contrary, TpA in YR··YR octanucleotide contexts have a strong preference for positive shift and rarely visit low twist or negative slide. Inspection of the trajectories suggests that this is probably due to a domino effect of h-bond proclivity so that depending on the base pairs flanking the R··Y hexanucleotide there is either an equally strong preference towards BII at ApG on the two strands, or the Watson strand BII state is favoured over the Crick, which is necessarily compensated by shifting the bases towards the major groove. Finally, remote sequence effects are present just in a few cases for R··R/Y··Y hexanucleotides and lead to a change in shift from the minor to the major groove, maintaining similar distributions of twist and slide (Figure 7). In summary, our results suggest that CTAG is one of the few tetranucleotides (amongst the unique 136) where next-to-nearest neighbours and beyond effects are observed, while in general, nearest neighbour models can accurately explain by ‘concatenation of tetranucleotides’ the described remote effects in longer sequences.

### Data mining of structural databases and genomic implications

**Figure 8.**
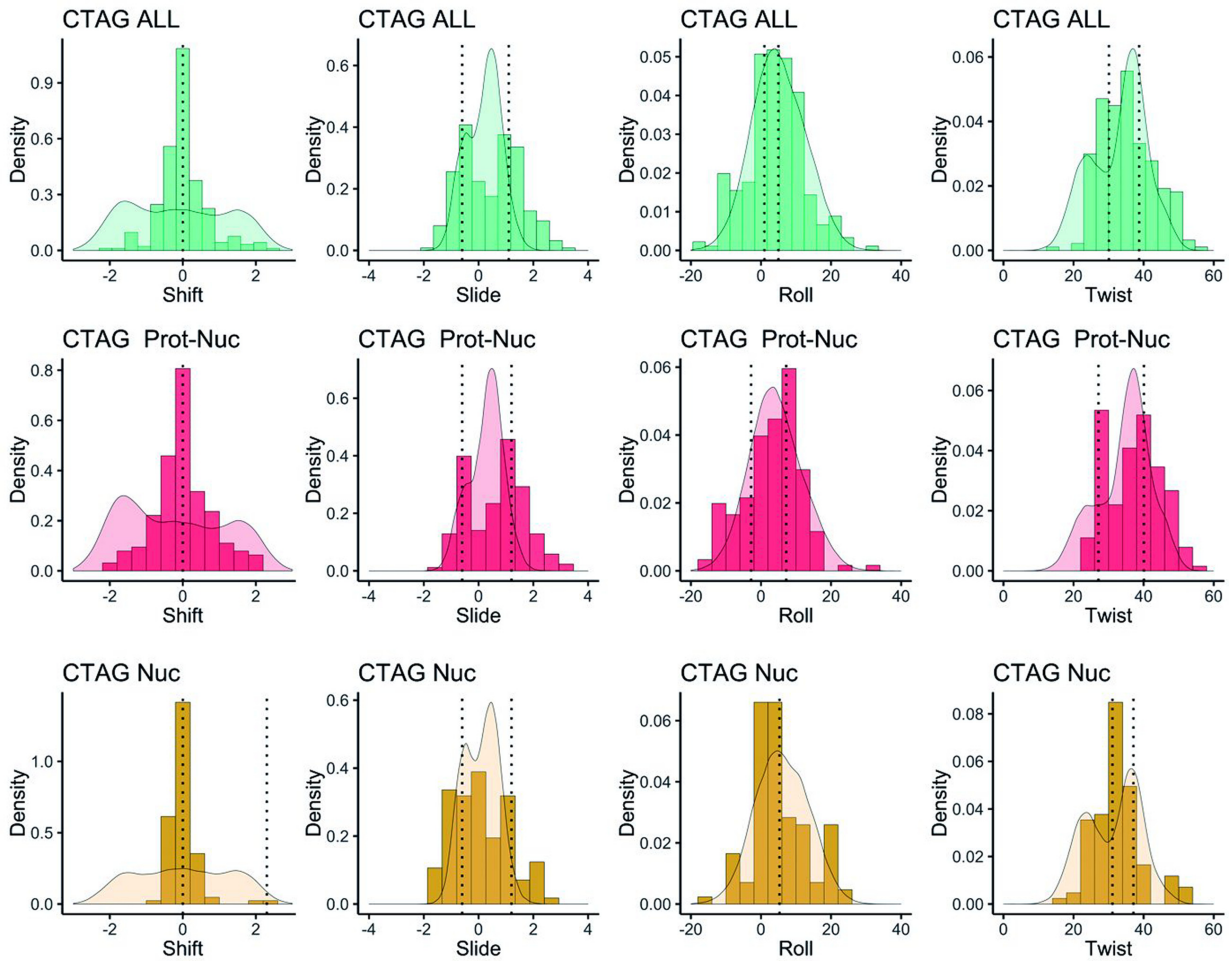
Normalized frequencies of shift, slide, roll and twist from MD meta-trajectory of representative hexanucleotides (G··C for free DNA; A··G, G··A, A··T and T··A for protein-bound DNA and their combination for all DNA structures) compared to those obtained from the data mining of the PDB for all structures containing DNA (first row), for Protein–DNA complexes (second row) and for isolated DNA structures (third row). The mean values of the BIC components of the experimental helical parameters data are shown as vertical dotted lines in each case.

We analysed the structures of DNA obtained experimentally (X-ray and NMR) and stored in the Protein Data Bank that contained the CTAG tetranucleotide sequence in order to validate our results. Only 106 occurrences of CTAG in naked DNA structures were found (some with small ligands or metal ions), and 160 occurrences in structures of protein–DNA complexes. Moreover, only a fraction of the tetranucleotide sequence space is covered (next-to-nearest neighbours), and barely any of the hexanucleotide context (octanucleotides of the type XpXpCpTpApGpXpX, where X = C, T, A, G) is found (Supplementary Table S3). This scarcity of data clearly limits the generality of the conclusions that could be derived from the data mining of the PDB, although a BIC analysis of the experimental structural parameters of TpA steps flanked by 5′C-3′G at least confirms that multi-modality is not a force field artefact (Supplementary Figure S9). PDB structures containing the CTAG tetranucleotide have values for the shift, slide, roll and twist helical parameters that cover the multi-modal distributions obtained in our trajectories, confirming our claims on the bimodal nature of slide and twist, with peaks in the distributions that fit well to our results (see Figure 8 and Supplementary Figure S9). For shift, TpA steps distribution displays peaks 2 Å towards both the minor or major groove in several protein-bound DNA structures, but the data on naked DNA seem to be insufficient to cover these deformations: there is a small peak at +2 Å, but highly underestimated compared to our results. Finally, roll has a broad distribution, similar to what we obtain from MD simulations, being bi-normal, but unimodal.

**Figure 9.**
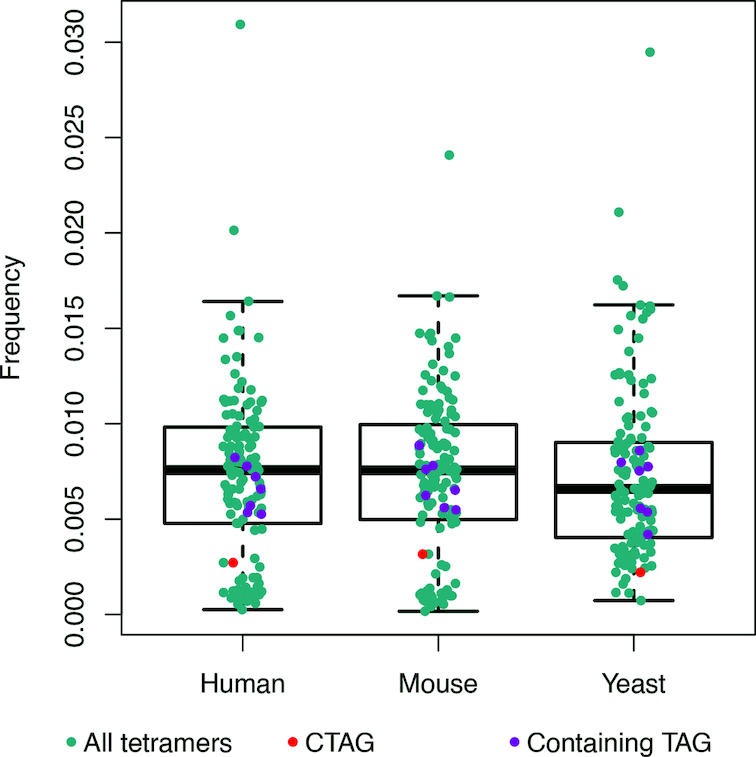
Frequency of each possible tetranucleotide in three different genomes. CTAG is marked in red, tetranucleotides containing TpApG (amber stop codon) are marked in violet and the rest are depicted in cyan.

All analyses performed in this work suggests that CTAG has really unique physical properties, which should provide the genome with a point of high flexibility and polymorphism. Very remarkably, CTAG is one of the lowest populated tetranucleotides in the analysed species (see Figure 9) appearing mainly on intergenic regions and very rarely on genes (Supplementary Figure S10). We further highlighted this by analysing comparatively, with and without including exons, all the tetranucleotides containing the trinucleotide TpApG (XTAG or TAGX, where X could be A, C, G or T), which is known as the amber stop codon. Our results still confirm the low rate of the CTAG tetranucleotide, even removing the TpApG stop codon (Supplementary Figure S11). Interestingly, this infrequent CTAG tetranucleotide is well conserved, which suggest that (i) despite being far from coding regions they are important for the functionality of the cell, or alternatively, (ii) they are easily accessible to the mismatch repairing machinery, avoiding the stabilization of mutations. The same conclusion can be reached from the analysis of cancer genomic data, which show that again CTAG is very rarely mutated in cancer (Supplementary Figure S12). The unusual physical properties of the CTAG tetranucleotide matches its unusual prevalence and distribution in the genome and its extreme resilience to somatic (cancer) mutations. It is tempting to believe that cell takes advantage of the unusual properties of CTAG as points of high flexibility that might help to fold chromatin.

## CONCLUSIONS

We present here an in-depth study of one of the most ‘structurally speaking’ polymorphic tetranucleotides found in B-DNA. The complete helical space of the CTAG tetranucleotide has been analysed by means of extensive molecular dynamics simulations and by data mining the Protein Data Bank, confirming its highly polymorphic behaviour at several helical parameters: shift, slide, twist and BI/BII. This confers to CTAG the possibility to exist in several different substates, being particularly flexible. We present here clear evidence that the type of substate displayed by CTAG in a given sequence context, and in consequence its dynamics, is sequence dependent, and fine-tuned by next-to-nearest neighbours and beyond. Based on the concerted and correlated movements of bases and backbone torsions for the described multi-modal degrees of freedom, and driven by the mechanical limitations imposed by DNA’s crankshaft motions, we were able to found a possible explanation on how structural information can travel almost half helical turn away from the central TpA step. This remote structural ‘connection’ allows the TpA step to ‘feel’ its sequence environment beyond the next-to-nearest neighbours, and eventually adopts a different substate if needed. Moreover, we found that previously described unconventional intra-molecular hydrogen bonds of the type C8H8-O3′ and C6H6-O3′ that link the movements of the bases with the torsions in the backbone, could be used as descriptors of such correlated motions. Finally, we established that although this highly flexible tetranucleotide is extremely under-represented in several genomes along the animal Kingdome, being mostly present in intergenic sequences, it has been preserved with a low rate of mutation implying a possible physical role for CTAG at the genomic level.

## Supplementary Material

Supplementary Data
