# Supplementary material for: Modulation of the helical properties of DNA: next-to-nearest neighbour effects and beyond
Source: Nucleic Acids Res. 2019 Apr 8;47(9):4418–30. doi: 10.1093/nar/gkz255 (PMC6511876; doi:10.1093/nar/gkz255)
Supplement: Supplementary Data [file gkz255_supplemental_file.pdf]

SUPPORTING INFORMATION

MODULATION OF THE HELICAL PROPERTIES OF DNA: NEXT-TO-NEAREST NEIGHBOUR EFFECTS AND BEYOND

Alexandra Balaceanu[1], Diana Buitrago[1], Jürgen Walther[1], Adam Hospital[1], Pablo D. Dans[1] and Modesto Orozco[1,2,*]

[1] Institute for Research in Biomedicine (IRB Barcelona), The Barcelona Institute of Science and Technology (BIST), 08028 Barcelona, Spain.
[2] Department of Biochemistry and Biomedicine, University of Barcelona, Barcelona, Spain.

* To whom correspondence should be addressed: Prof. Modesto Orozco, Tel: +34 93 403 7155, Fax: +34 93 403 7157, Email: modesto.orozco@irbbarcelona.org.

SUPPORTING METHODS

The choice of sequences. We built a library of 40 different 16 bp oligomer sequences with a middle $d(CpTpApG)_2$ that cover the entire hexanucleotide space featuring a XpCpTpApGpX sequence pattern (X stands for any nucleotide) as well as all possible pyrimidine(Y)/purine(R) combinations at the octanucleotide level in several (>3) repeats.

System preparation and MD simulations. All the sequences were prepared with the leap program of AMBERTOOLS 16 (1) and simulated using pmemd.cuda code (2). Following the ABC protocol (3), canonical duplexes were generated using Arnott B-DNA fiber parameters (4), and solvated by a truncated octahedral box with a minimum distance of 10 Å between DNA and the closest face of the box.

Simulations were run using parmbsc1 force-field, SPC/E water model (5) and 150 mM concentration of K^+Cl^- salt using Smith/Dang parameters (6–8). Systems were optimized and equilibrated as described in our previous works, and simulated for at least 500 ns and up to 10 μs in the NPT ensemble, using Particle-Mesh Ewald

corrections (2, 9) and periodic boundary conditions. SHAKE was used to constrain bonds involving hydrogen (10), allowing 2 fs integration step. All the trajectories and the associated analysis are accessible in the BigNAsim portal: https://mmb.irbbarcelona.org/BIGNASim/.

Analysis of Molecular Dynamics trajectories. All the trajectories were processed with the *cpptraj* module of the AMBERTOOLS 16 package (1), and the NAFlex server (10) for standard analysis. DNA helical parameters and backbone torsion angles were measured and analysed with the CURVES+ and CANAL programs (11), following the standard ABC conventions (3). The CANION module from Curves+ (12) was used to determine the position of cations in curvilinear cylindrical coordinates for each snapshot of the simulations with respect to the instantaneous helical axis. We obtained and analysed the ion distribution in one- (R, D, A) and two-dimensional (RA, DA, DR) curvilinear cylindrical coordinates at the central tetranucleotide sequence. Duplexes were named following the Watson strand (*e.g.* CTAG stands for (CTAG)·(CTAG)). The letters R, Y and X stand for a purine a pyrimidine or any base respectively, while X·X and XX represent a base pair and base-pair step respectively. Base pairs flanking the CTAG were denoted using two dots to represent the central tetrad (e.g. R··Y).

The Essential Modes of generic TpA in helical space. We performed Principal Component Analysis (PCA) of the 18 intra- and inter- base-pair parameters that define all degrees of freedom of the central TpA step in a rigid-base model. Before calculating the covariance matrix in helical space, its entries had to be made dimensionally uniform, so all rotational degrees of freedom were scaled by a factor of 10.6 (13). The covariance was calculated from the joint equilibrated trajectories of all 40 sequences taken at every 100 ps. The first 3 Principal Components, which explain ~60% of the total variance, have their largest projections on a subset of 8 of the original 18 helical parameters. These 3 PCs were used to perform multidimensional clustering in the essential helical space using the mclust package of R. The clustering is performed using the optimal model according to Bayesian Information Criterion (BIC) for an expectation-minimization (EM) algorithm initialized by hierarchical clustering for parameterized Gaussian mixture models.

Distributions of helical parameters that guide specific sequence dependence. The helical parameters that showed the highest variability across trajectories of different sequences were identified using Principal Component Analysis (PCA) of the 18 intra- and inter base pair parameters that define all degrees of freedom of the central TpA step in a rigid-base model. The first 3 Principal Components, which explain ~60% of the total variance have their largest projections on a subset of 8 of the original 18 helical parameters. The Bayesian Information Criterion (BIC) (14, 15) was used, limiting the analysis to either two or three components to determine the number of normal functions needed to meaningfully represent the appearance

of possible substates in the shift, slide, roll and twist 1D distributions of the joint trajectory of all sequences. The normal distributions obtained from the BIC decomposition were compared to the distributions of the same parameters obtained after the multivariate clustering (into 3 clusters) of the first 3 PCs.

From the eight parameters identified from the PCA as accounting for the most variance, six are non-collinear in the essential helical space, namely the shift, slide and twist of TpA bps, the buckle and propeller twist of dT and the buckle of dA. The distributions of the subset of these 6 parameters were used to evaluate the similarity between central TpA steps in different oligonucleotide sequences using the Kullback-Leibler (KL) divergence theorem. For each pair of oligomers we calculated the symmetrized values of the KL divergence and then applied hierarchical cluster analysis using Ward's clustering criterion (16), where the dissimilarities are squared before cluster updating (17) in order to identify specific sequence effects on TpA helical space flexibility.

The 4-state model of TpA dynamics. The 3D and 2D distributions of these three parameters and their paired combinations, respectively, in the meta-trajectory have also been calculated and they show a clear preference of the TpA to occupy one of four states in the Shift-Slide-Twist space. In fact, the states of the 3 helical parameters that display polymorphisms are highly inter-dependent, as shown in the 2- and 3- dimensional distribution plots. The 3 most populated states in the twist-slide-shift space, when considering the entire meta-trajectory of all oligonucleotides, are: High Twist/Positive Slide/Negative Shift (HPN), High Twist/Positive Slide/Positive Shift (HPP), and Low Twist/Negative Slide/Zero Shift (LNZ). In order to capture and better understand these effects, we filtered the meta-trajectory into 3 sub-trajectories corresponding to the 3 states, removing all frames that did not belong to any of these. We compared the distribution of helical parameters beyond the next-to-nearest neighbours (octanucleotide level) in both directions ("-" sign for moving towards the 5' direction on the Watson strand and "+" sign for the 3' direction) between the 3 substate-trajectories and found significant effects in the neighbouring shift, slide and twist. We also compared up to the octanucleotide level, backbone torsions, sugar puckering, and glycosidic torsions.

Breaking down the twist, slide and shift contributions to the distal sequence effects, we calculate the Pearson's correlations of these parameters at TpA to the helical parameters at one and two levels away from TpA in each direction and the point biserial correlations to the backbone torsion (zeta – categorized in trans and gauche-), sugar pucker (categorized into South and North) and glycosidic torsion (categorized into Anti and High Anti).

Equilibrium distributions of inter base pair helical parameters at the TpA step vary beyond next-to-nearest neighbours. BIC (Bayesian Information Criterion) was used to distinguish between the normal (one Gaussian) or multi-normal (a mixture of two or more Gaussians) nature of the distributions of TpA helical parameters (14, 15).

Since for each individual trajectory, the BIC decomposition assign the same number of Gaussians (1, 2 and 3) in the respective helical parameters (roll, twist/slide and shift, respectively) and the peaks of the distributions are consistent thought the set of oligomers, we compare the propensities of each Gaussian of the individual trajectories with the total average propensity per peak, assigning them to one of three ranges: mean – sd, mean + sd and within this interval, in order to identify large deviations in population imposed by sequence.

Correlation between twist and zeta states. As previously analysed in depth for the CpG case, we found strong correlations between the twist state and the BI/BII backbone state at the 3' side of the TpA step on both Watson and Crick strands. The backbone state was defined by discretizing the zeta torsion sub-states into trans (180 ± 40 degrees – associated with a backbone in BII), gauche positive (60 ± 40 degrees – extremely infrequent) and gauche negative (300 ± 40 degrees – associated with a backbone in BI). Just like in the CpG case, a low twist state was found to usually be coupled with BII transitions at both 3' junctions.

Correlation between twist and C-H··O3' hydrogen bond. Relying on strong evidence from previous studies (18, 19) of almost perfect correlation between backbone state and the formation of base to backbone hydrogen bonds, we looked at the correlation between twist state at the TpA step and hydrogen bond formation beyond the next-to-nearest neighbours. We found, as expected, a dependency of 3' side adjacent bond formation to twist state that perfectly mirrors that of the backbone state. But we also discovered an insightful sequential anti-correlation of bond formation from one step to the next that is also highly dependent on sequence, which favours the formation of one or the other.

Stacking and Base-pairing strength. In order to estimate the strength of stacking at the TpA step we calculated a Stacking Factor based on the distance between the centres of mass of DT and DA, and the angle between the two planes of the bases, defined as (20):

$$\xi = \frac{r_M}{S(\alpha)}$$

$$S(\alpha) = e^{-\alpha} + e^{-(\alpha-\pi)^4} + 0.1e^{-(\alpha-0.5\pi)^4}$$

where r_M is the distance between the two centres of mass and α the angle between the base planes. We calculated the Stacking Factors separately for the major 3 of the 4 states in twist/slide/shift space defined above to determine the stabilizing factors of the highly preferred states.

Database Analysis of structural features. We retrieved high resolution (< 3Å) structures of double stranded DNA containing the CTAG tetrad and distinguished between the protein-bound and free DNA structures. We compared helical parameter distributions and components of BIC analysis between the database structures and out results. We paid special attention to the sequence context bias found in the database and performed the comparison to the meta-trajectory from simulations containing the same hexanucleotide environments centred at TpA.

Database Analysis of genomic properties. Prevalence of CTAG in the genomes of *H. sapiens* (hg19), *E. coli* (NC_000913.3) and *S. cerevisiae* (sacCer3) was computed, finding low occurrence compared to other tetranucleotides (less than 0.5% in the three species). Occurrences of this tetranucleotide were then mapped, using Homer software (21), to the annotated regions of each organism obtained from UCSC and compared to the overall frequency of each annotation type. CTAG is enriched at intergenic regions in *H. sapiens* and *E. coli*, but not in *S. cerevisiae* probably due to the low number of intergenic regions in this organism (less than 2.5% compared to more than 20% in the other two). To evaluate resilience to mutation, the frequency of mutations for each tetranucleotide (normalised by tetranucleotide frequency) along the genome in 30 different cancer types (22) was computed. SNPs in human genome were retrieved from Ensembl Variation database (23) and were mapped to each tetranucleotide to compute normalized SNP frequency per tetranucleotide.

SUPPORTING TABLES

Table S1. Sequence library used to study CTAG polymorphisms, number of replicas and simulation time.

Num.	Sequence	Simulation time	Num.	Sequence	Simulation time
1	CGTCGGCTAGCCGAGC	500 ns	21	CGGAGACTAGACTCGC	500 ns
2	CGTCTCCTAGGAGAGC	500 ns	22	CGGAGACTAGCCTCGC	500 ns
3	CGAAAACTAGAAAAGC	500 ns	23	CGGAGACTAGGCTCGC	6 µs
4	CGAAAACTAGTTTTGC	500 ns	24	CGGAGACTAGTCTCGC	6 µs
5	CGATATCTAGATATGC	500 ns	25	CGGAGCCTAGACTCGC	500 ns
6	CGTATACTAGTATAGC	2 x 500 ns	26	CGGAGCCTAGCCTCGC	2 x 500 ns
7	CGGGGGCTAGGGGGGC	500 ns	27	CGGAGCCTAGGCTCGC	500 ns
8	CGGGGGCTAGCCCCGC	500 ns	28	CGGAGGCTAGACTCGC	500 ns
9	CGGCGCCTAGGCGCGC	500 ns	29	CGGAGGCTAGCCTCGC	6 µs
10	CGCGCGCTAGCGCGGC	500 ns	30	CGGAGTCTAGACTCGC	2 x 500 ns
11	CGTCTACTAGAGAGGC	500 ns	31	CGCTAGCTAGCTAGGC	4 x 500 ns
12	CGTCTACTAGCGAGGC	2 x 500 ns	32	CGATATCTAGAAATGC	2 µs
13	CGTCTACTAGGGAGGC	6 µs	33	CGGAGCCTAGAATCGC	2 µs
14	CGTCTACTAGTGAGGC	2 x 500 ns	34	CGGCGCCTAGGGGCGC	2 µs
15	CGTCTCCTAGAGAGGC	2 x 500 ns	35	CGGAGGCTAGCATCGC	2 µs
16	CGTCTCCTAGCGAGGC	500 ns	36	CGAAAACTAGTATAGC	2 µs
17	CGTCTCCTAGGGAGGC	500 ns	37	CGCTAGCTAGCGAGGC	2 µs
18	CGTCTGCTAGAGAGGC	6 µs	38	CGTCTGCTAGACAGGC	2 µs
19	CGTCTGCTAGCGAGGC	9 µs	39	CGAATCCTAGATAAGC	2 µs
20	CGTCTTCTAGAGAGGC	500 ns	40	CGGACACTAGCGTCGC	2 µs

Table S2. Pearson correlation coefficients of Shift, Slide and Twist at TpA with flanking bps parameters and selected backbone torsions up to next-to-nearest neighbours.

		**Shift at TA**	**Slide at TA**	**Twist at TA**		**Shift at TA**	**Slide at TA**	**Twist at TA**
**-2**	Shift	0.06	0.002	0.025	zetaW	-0.067	-0.063	-0.123
	Slide	0.157	0.149	0.206	zetaC	-0.471	-0.286	-0.421
	Rise	-0.052	-0.022	-0.086	phaseW	-0.130	-0.023	-0.073
	Tilt	0.086	0.031	0.051	phaseC	-0.061	-0.079	-0.110
	Roll	0.001	0.043	0.038	chiW	0.018	0.002	0.025
	Twist	0.089	0.051	0.021	chiC	-0.074	-0.042	-0.057
**-1**	Shift	-0.607	-0.149	-0.257	zetaW	-0.454	-0.098	-0.217
	Slide	-0.298	0.089	-0.094	zetaC	0.753	0.295	0.536
	Rise	0.028	-0.089	-0.109	phaseW	-0.425	0.006	-0.105
	Tilt	-0.12	0.057	-0.11	phaseC	0.111	0.102	0.090
	Roll	0.002	0.178	0.157	chiW	-0.140	-0.027	-0.058
	Twist	-0.223	-0.263	-0.453	chiC	0.107	0.173	0.153
**Central TpA step**								
**+1**	Shift	-0.607	0.192	0.306	zetaW	-0.736	0.340	0.589
	Slide	0.201	0.098	-0.078	zetaC	0.456	-0.166	-0.260
	Rise	0.017	-0.08	-0.114	phaseW	-0.157	0.130	0.103
	Tilt	-0.104	-0.047	0.12	phaseC	0.431	-0.045	-0.144
	Roll	-0.045	0.176	0.173	chiW	-0.206	0.186	0.170
	Twist	0.232	-0.25	-0.455	chiC	0.166	-0.022	-0.053
**+2**	Shift	0.185	-0.084	-0.148	zetaW	0.547	-0.332	-0.487
	Slide	-0.251	0.195	0.271	zetaC	0.023	-0.023	-0.061
	Rise	0.09	-0.04	-0.103	phaseW	0.020	-0.072	-0.076
	Tilt	0.156	-0.091	-0.125	PhaseC	0.085	-0.004	-0.054
	Roll	0.012	0.044	0.039	chiW	0.019	-0.012	-0.018
	twist	-0.095	0.079	0.067	chiC	-0.067	0.006	0.024

Table S3. Number and frequency of unique occurrences of hexanucleotides containing central CTAG in the PDB database.

Type	Hexanucleotide Context	No. structures	Frequency
**Naked DNA structures**	G··C	15	0.54
	A··T	5	0.18
	T··A	3	0.11
	C··G	2	0.07
	T··T	2	0.07
	T··C	1	0.04
**Protein-DNA complexes**	A··G	30	0.31
	G··A	30	0.31
	T··A	11	0.11
	A··T	8	0.08
	G··G	7	0.07
	C··G	5	0.05
	A··A	2	0.02
	G··C	2	0.02
	A··C	1	0.01
	T··G	1	0.01

SUPPORTING FIGS.

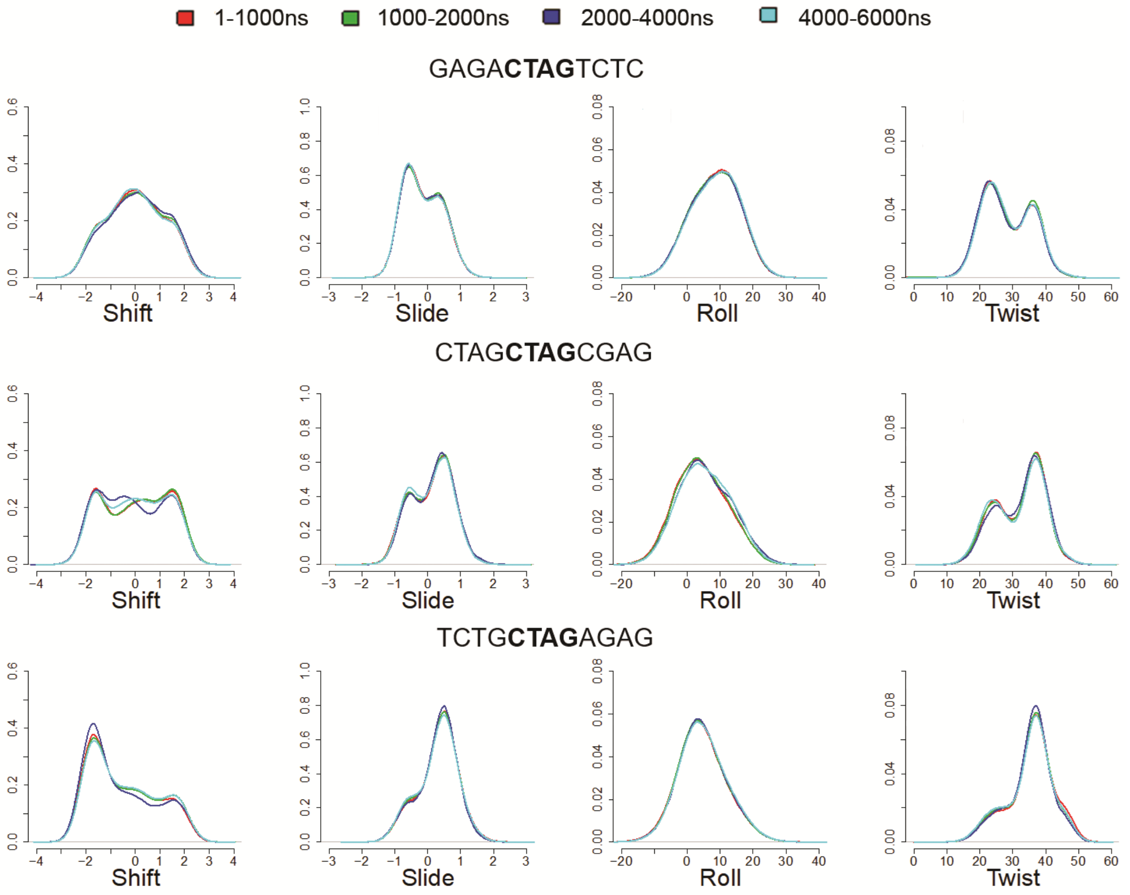

Fig. S1. Normalized frequencies of the shift, slide, roll and twist helical parameters for 3 selected sequences, whose trajectories were extended to 6 μs to check for convergence. Four distributions were computed for each helical parameter using segments of 1,000 or 2,000 ns.

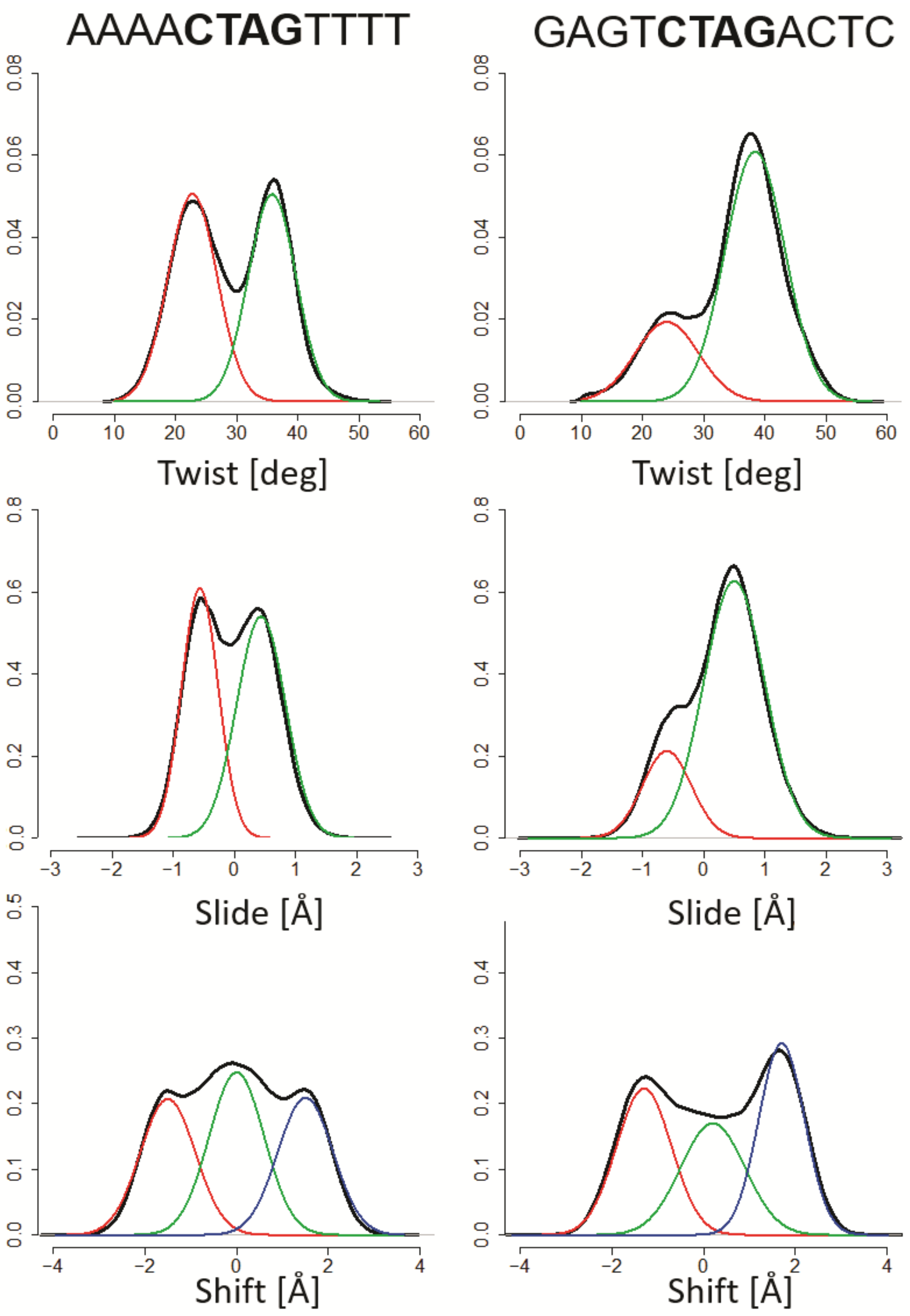

Fig. S2. Normalised frequencies of the shift, slide, and twist helical parameters for 2 selected sequences showing clear next-to-nearest neighbour effects, which could be appreciated from the change in the relative populations of the bi- and tri-normal distributions.

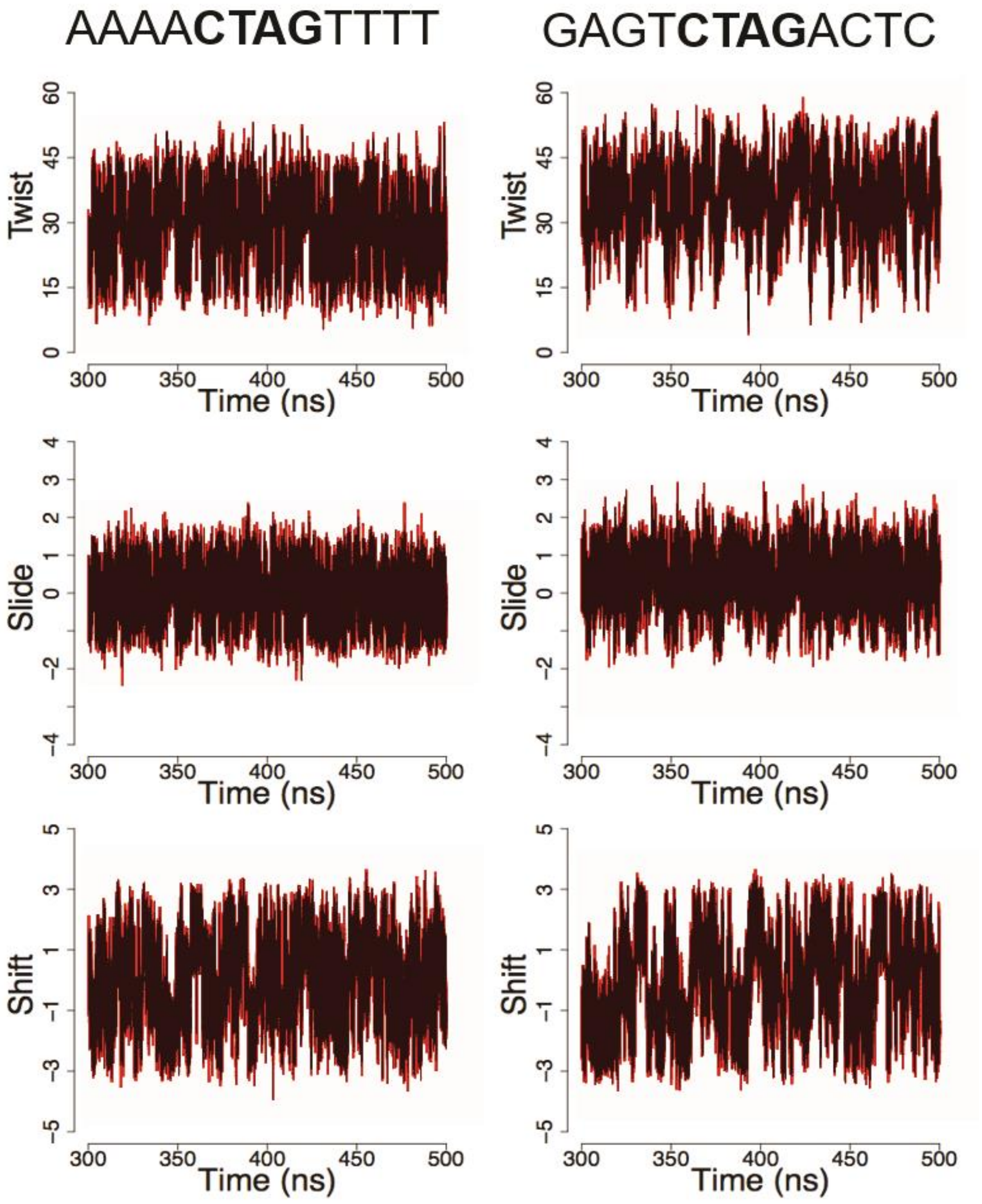

Fig. S3. Time evolution (500 ns) of shift, slide and twist for two selected sequences, showing the fast and reversible inter-conversion between high and low substates.

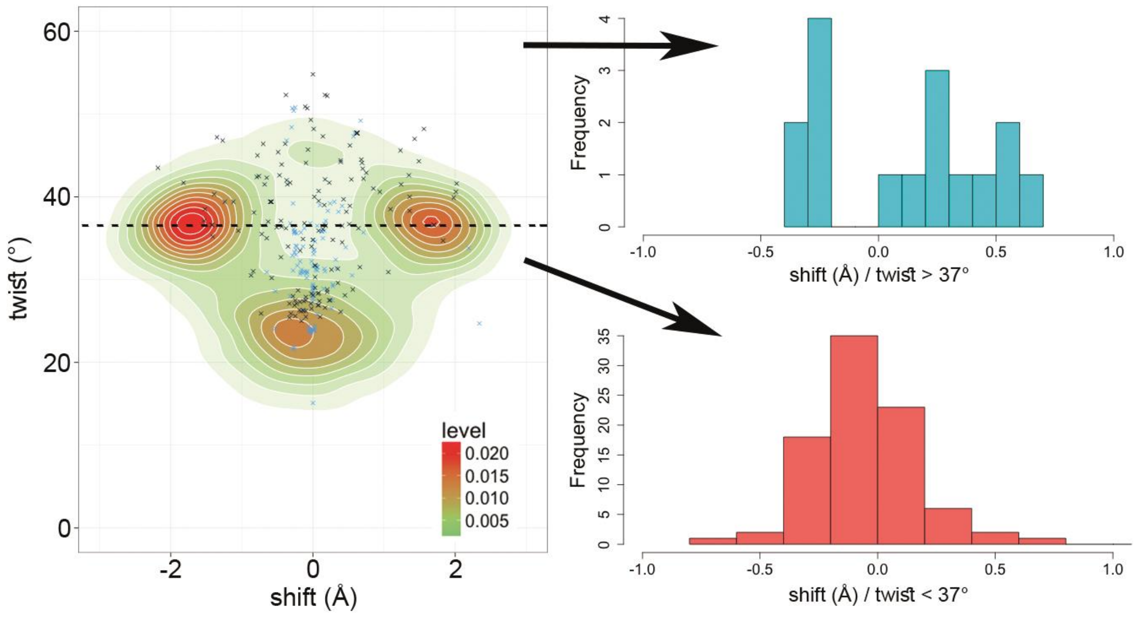

Fig. S4. 2D counts in the shift-twist plane from MD simulations at the central TpA step. In the 2D density plots experimental structures from the PDB (see Supp. Methods) were added as black crosses (Protein-DNA complexes), or blue crosses (isolated DNA). We divided the plane between high twist (> 37°), and low twist (< 37°) and analysed the shift distribution for these two cases.

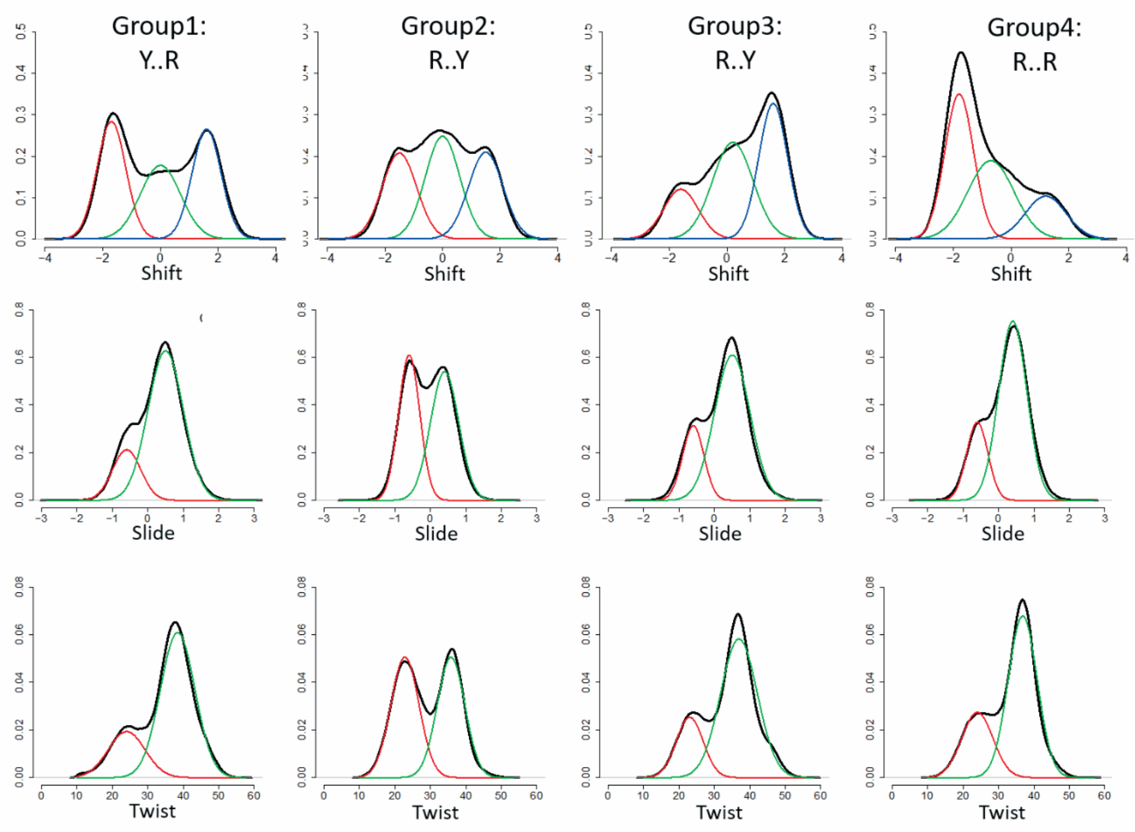

Fig. S5. Normalized frequencies for shift, slide and twist (black line), and the BIC decomposition in Gaussians (red, green, and blue lines), showing the behaviour of the clusters obtained in the dendrogram of Fig. 5.

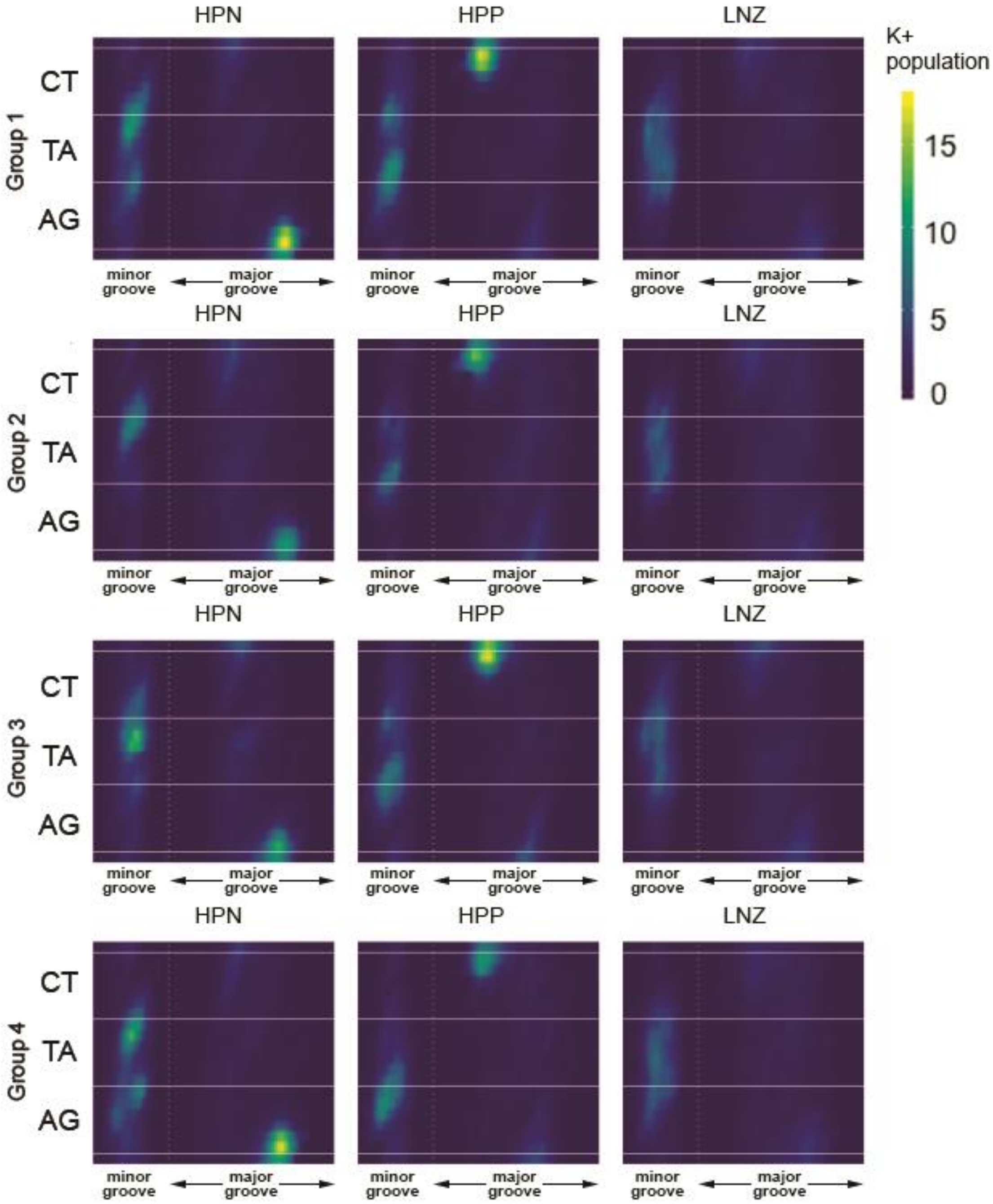

Fig. S6. Population of K+ ions inside the major and minor groove for bps CpT, TpA, and ApG in each of the three major states based on twist/slide/shift values at TpA.

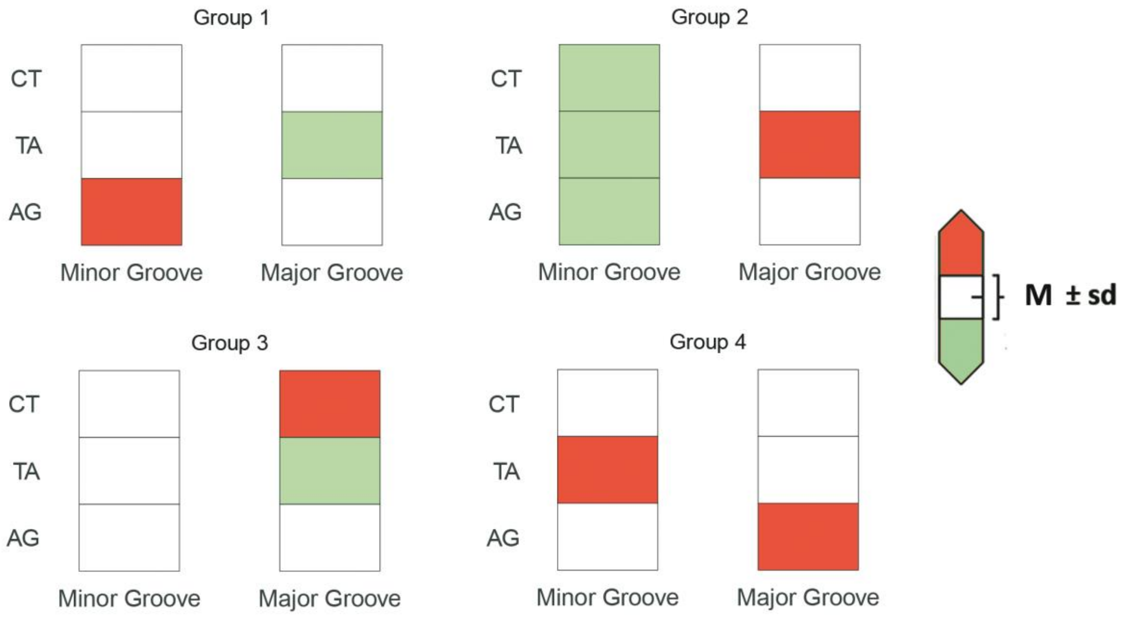

Fig. S7. Relative ion populations of cluster representatives in the minor and major groove at the CTAG tetranucleotide. Comparison to the global average ion populations per region.

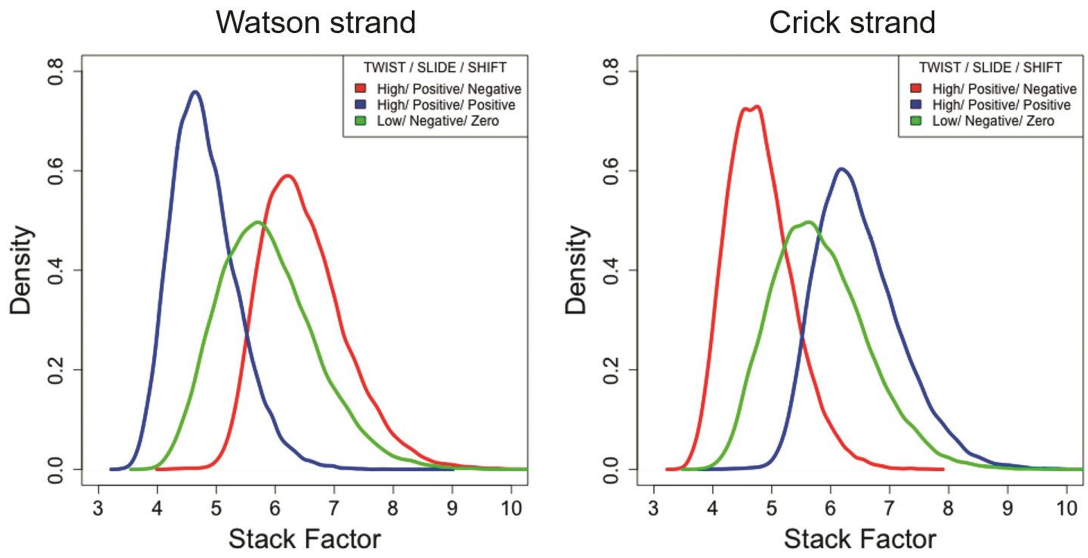

Fig. S8. Distributions of stacking coordinate at the TpA step on both Watson (left) and Crick (right) strands in the three main configurations of the bps in helical space.

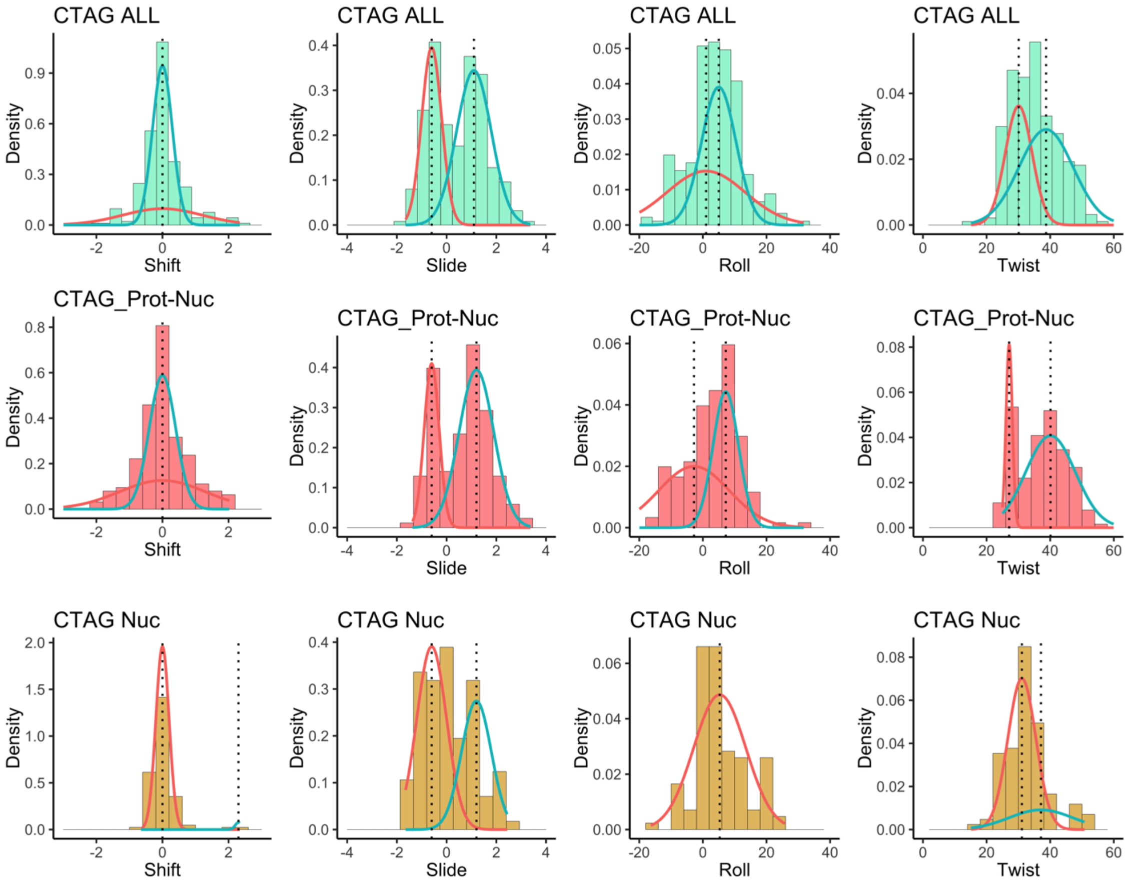

Fig. S9. Normalized frequencies of shift, slide, roll and twist at TpA obtained from the data mining of the PDB for all structures containing CTAG according to BIC analysis: all DNA (FIRST ROW), Protein-DNA complexes (SECOND ROW), and isolated DNA structures (THIRD ROW).

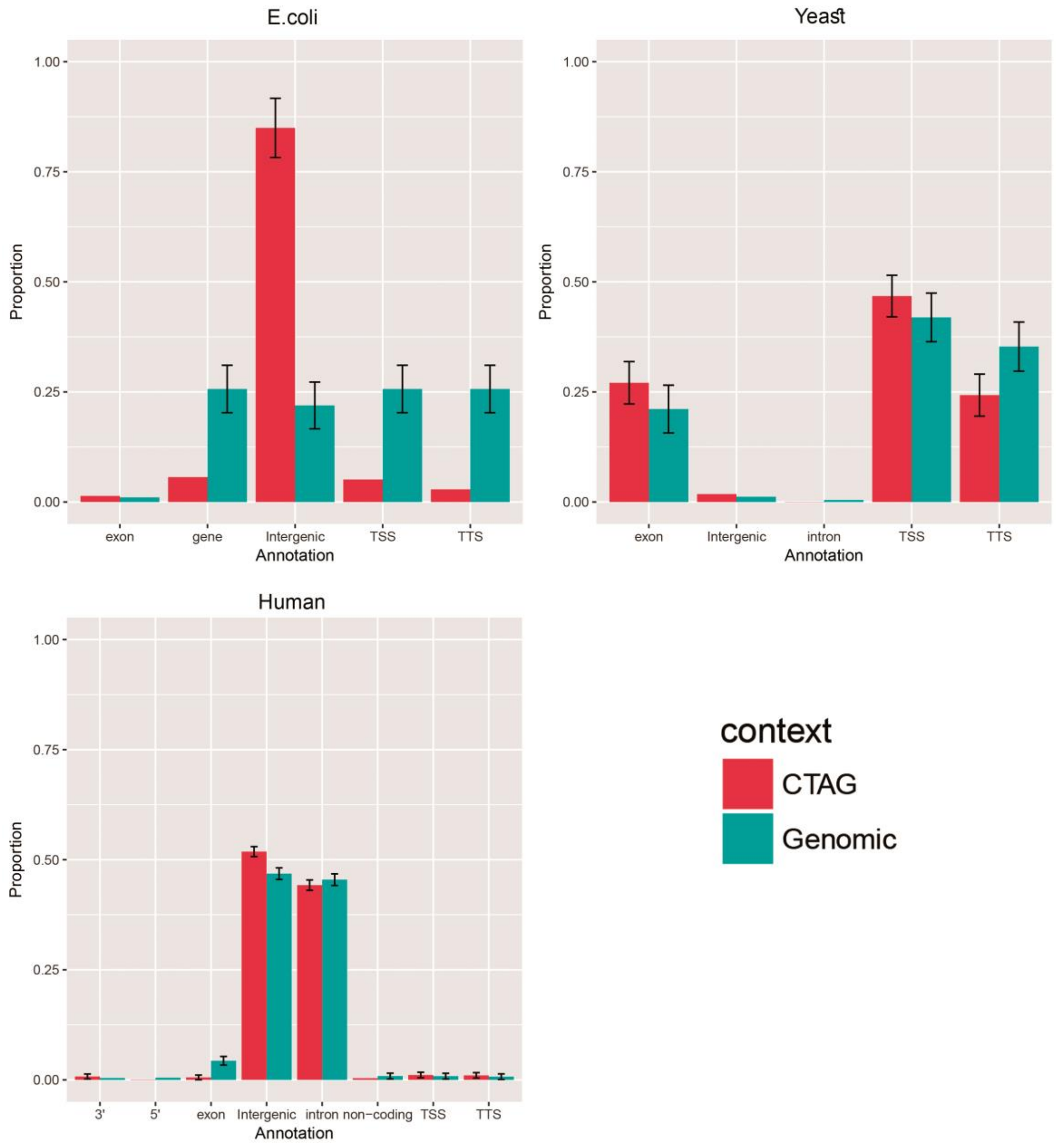

Fig. S10. Occurrence of CTAG in different genomic regions. Length of each annotation type is shown to evaluate significance of enrichment per region type.

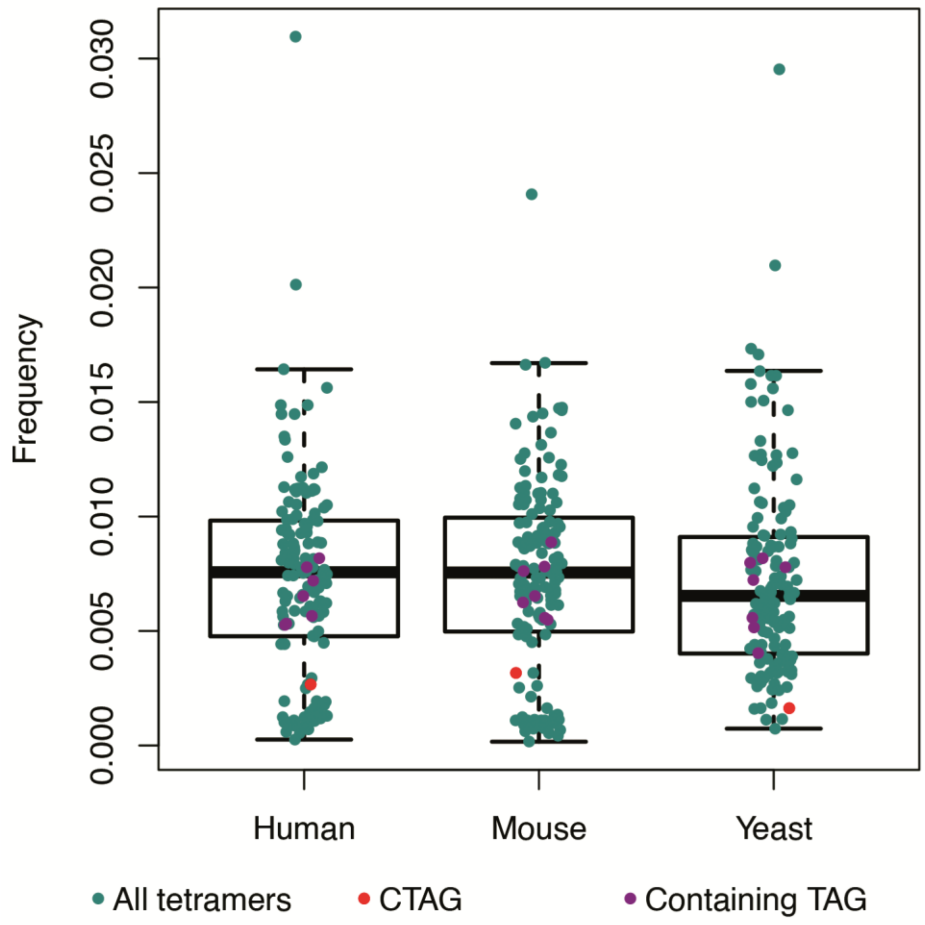

Fig. S11. Frequency of each possible tetranucleotide in 3 different genomes. CTAG is marked in red, tetranucleotides containing TpApG (all but the amber stop codon) are marked in violet, and the rest are depicted in cyan. Note that this analysis doesn't includes exons.

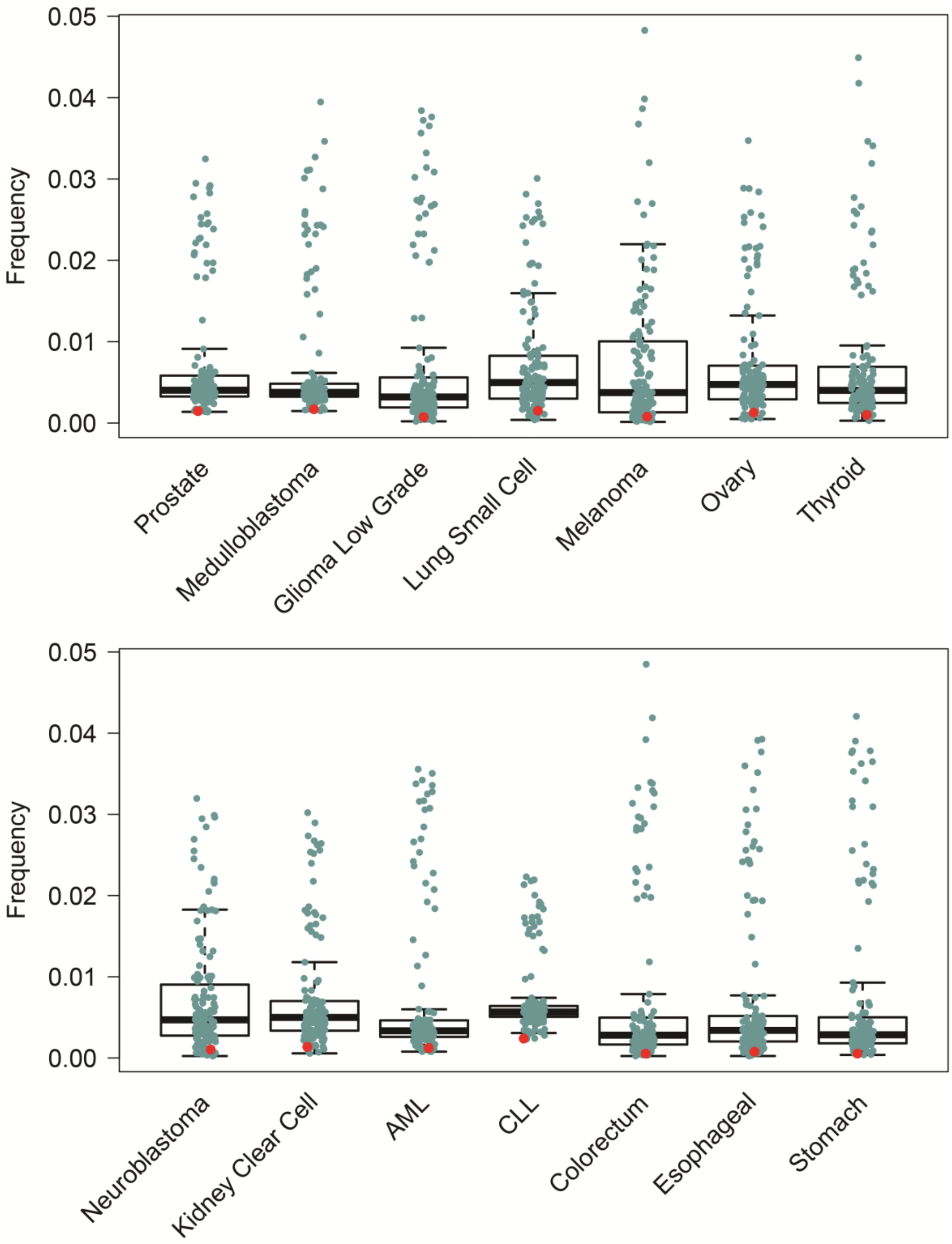

Fig. S12. Frequency of mutations for each tetranucleotide along the genome for several cancer types, normalised by genome-wide tetranucleotide occurrence. CTAG is marked in red.

SUPPORTING REFERENCES

1. D.A. Case, R.M. Betz, D.S. Cerutti, T.E. Cheatham, III, T.A. Darden, R.E. Duke, T.J. Giese, H. Gohlke, A.W. Goetz, N. Homeyer, S. Izadi, P. Janowski, J. Kaus, A. Kovalenko, T.S. Lee, S. LeGrand, P. Li, C. Lin, T. Luchko, R. Luo, B. Madej, D. Mermelstein,L.X. and P.A.K. (2016) AMBER 2016.
2. Le Grand,S., Götz,A.W. and Walker,R.C. (2013) SPFP: Speed without compromise—A mixed precision model for GPU accelerated molecular dynamics simulations. *Comput. Phys. Commun.*, **184**, 374–380.
3. Pasi,M., Maddocks,J.H., Beveridge,D., Bishop,T.C., Case,D.A., Cheatham,T., Dans,P.D., Jayaram,B., Lankas,F., Laughton,C., *et al.* (2014) µABC: A systematic microsecond molecular dynamics study of tetranucleotide sequence effects in B-DNA. *Nucleic Acids Res.*, **42**, 12272–12283.
4. Arnott,S. and Hukins,D.W.L. (1973) Refinement of the structure of B-DNA and implications for the analysis of X-ray diffraction data from fibers of biopolymers. *J. Mol. Biol.*, **81**, 93–105.
5. Berendsen,H.J.C., Grigera,J.R., Straatsma,T.P., Grigera,J.R., Straatsma,T.P., Berendsen,H., Grigera,J., Straatsma,T., Grijera,J., Berendsen,H.J.C., *et al.* (1987) The missing term in effective pair potentials. *J. Phys. Chem.*, **91**, 6269–6271.
6. Smith,D.E. and Dang,L.X. (1994) Computer simulations of NaCl association in polarizable water. *J. Chem. Phys.*, **100**, 3757–3766.
7. Dang,L.X. (1995) Mechanism and Thermodynamics of Ion Selectivity in Aqueous Solutions of 18-Crown-6 Ether: A Molecular Dynamics Study. *J. Am. Chem. Soc.*, **117**, 6954–6960.
8. Dang,L.X. and Kollman,P.A. (1995) Free Energy of Association of the K+:18-Crown-6 Complex in Water: A New Molecular Dynamics Study. *J. Phys. Chem.*, **99**, 55–58.
9. Darden,T., York,D. and Pedersen,L. (1993) Particle mesh Ewald: An $N \cdot \log(N)$ method for Ewald sums in large systems. *J. Chem. Phys.*, **98**, 10089–10092.
10. Ryckaert,J.-P., Ciccotti,G. and Berendsen,H.J. (1977) Numerical integration of the cartesian equations of motion of a system with constraints: molecular dynamics of n-alkanes. *J. Comput. Phys.*, **23**, 327–341.
11. Lavery,R., Moakher,M., Maddocks,J.H., Petkeviciute,D. and Zakrzewska,K. (2009) Conformational analysis of nucleic acids revisited: Curves+. *Nucleic Acids Res.*, **37**, 5917–5929.
12. Pasi,M., Maddocks,J.H. and Lavery,R. (2015) Analyzing ion distributions around DNA: sequence-dependence of potassium ion distributions from microsecond molecular dynamics. *Nucleic Acids Res.*, **43**, 2412–23.
13. Dršata,T. and Lankaš,F. (2013) Theoretical models of DNA flexibility. *Wiley Interdiscip. Rev. Comput. Mol. Sci.*, **3**, 355–363.
14. Schwarz,G. (1978) Estimating the Dimension of a Model. *Ann. Stat.*, **6**, 461–464.
15. Kass,R.E. and Raftery,A.E. (1995) Bayes Factors. *J. Am. Stat. Assoc.*, **90**, 773–795.
16. Ward,J.H. (1963) Hierarchical Grouping to Optimize an Objective Function. *J. Am.*

Stat. Assoc., **58**, 236–244.
17. Murtagh,F. and Legendre,P. (2014) Ward's Hierarchical Agglomerative Clustering Method: Which Algorithms Implement Ward's Criterion? *J. Classif.*, **31**, 274–295.
18. Dans,P.D., Faustino,I., Battistini,F., Zakrzewska,K., Lavery,R. and Orozco,M. (2014) Unraveling the sequence-dependent polymorphic behavior of d(CpG) steps in B-DNA. *Nucleic Acids Res.*, **42**, 11304–11320.
19. Balaceanu,A., Pasi,M., Dans,P.D., Hospital,A., Lavery,R. and Orozco,M. (2017) The Role of Unconventional Hydrogen Bonds in Determining BII Propensities in B-DNA. *J. Phys. Chem. Lett.*, **8**.
20. Jafilan,S., Klein,L., Hyun,C. and Florián,J. (2012) Intramolecular Base Stacking of Dinucleoside Monophosphate Anions in Aqueous Solution. *J. Phys. Chem. B*, **116**, 3613–3618.
21. Heinz,S., Benner,C., Spann,N., Bertolino,E., Lin,Y.C., Laslo,P., Cheng,J.X., Murre,C., Singh,H. and Glass,C.K. (2010) Simple Combinations of Lineage-Determining Transcription Factors Prime cis-Regulatory Elements Required for Macrophage and B Cell Identities. *Mol. Cell*, **38**, 576–589.
22. Alexandrov,L.B., Nik-Zainal,S., Wedge,D.C., Aparicio,S.A.J.R., Behjati,S., Biankin,A. V., Bignell,G.R., Bolli,N., Borg,A., Børresen-Dale,A.-L., *et al.* (2013) Signatures of mutational processes in human cancer. *Nature*, **500**, 415–421.
23. Zerbino,D.R., Achuthan,P., Akanni,W., Amode,M.R., Barrell,D., Bhai,J., Billis,K., Cummins,C., Gall,A., Girón,C.G., *et al.* (2018) Ensembl 2018. *Nucleic Acids Res.*, **46**, D754–D761.
